# CDC50A is required for aminophospholipid transport and cell fusion in mouse C2C12 myoblasts

**DOI:** 10.1242/jcs.258649

**Published:** 2021-10-19

**Authors:** Marta Grifell-Junyent, Julia F. Baum, Silja Välimets, Andreas Herrmann, Coen C. Paulusma, Rosa L. López-Marqués, Thomas Günther Pomorski

**Affiliations:** ^1^ Department of Molecular Biochemistry, Faculty of Chemistry and Biochemistry, Ruhr University Bochum, Bochum, Germany; ^2^ Department of Plant and Environmental Sciences, University of Copenhagen, Thorvaldsensvej 40, DK-1871 Frederiksberg C, Denmark; ^3^ Institut für Biologie, Molekulare Biophysik, IRI Life Sciences, Humboldt-Universität zu Berlin, Invalidenstrasse 42, 10115 Berlin, Germany; ^4^ Amsterdam UMC, University of Amsterdam, Tytgat Institute for Liver and Intestinal Research, Amsterdam Gastroenterology and Metabolism, Meibergdreef 9, 1105 AZ Amsterdam, The Netherlands

**Keywords:** Phospholipid, Aminophospholipid translocase, P4-ATPase, Myogenesis, Skeletal myoblasts

## Abstract

Myoblast fusion is essential for the formation of multinucleated muscle fibers and is promoted by transient changes in the plasma membrane lipid distribution. However, little is known about the lipid transporters regulating these dynamic changes. Here, we show that proliferating myoblasts exhibit an aminophospholipid flippase activity that is downregulated during differentiation. Deletion of the P4-ATPase flippase subunit CDC50A (also known as TMEM30A) results in loss of the aminophospholipid flippase activity and compromises actin remodeling, RAC1 GTPase membrane targeting and cell fusion. In contrast, deletion of the P4-ATPase ATP11A affects aminophospholipid uptake without having a strong impact on cell fusion. Our results demonstrate that myoblast fusion depends on CDC50A and may involve multiple CDC50A-dependent P4-ATPases that help to regulate actin remodeling.

This article has an associated First Person interview with the first author of the paper.

## Teaser

**Summary:** Myoblasts exhibit an aminophospholipid flippase activity that relies on CDC50A-dependent P4-ATPases. Loss of CDC50A compromises actin remodeling, RAC1 membrane targeting and cell fusion.

## INTRODUCTION

Phospholipid flip-flop in the plasma membrane of eukaryotic cells is highly regulated to maintain a non-random lipid distribution between the two leaflets of the plasma membrane. In general, the aminophospholipids phosphatidylserine (PS) and phosphatidylethanolamine (PE) are largely confined to the cytosolic leaflet, while sphingolipids (i.e. sphingomyelin and glycosphingolipids) are enriched in the exoplasmic leaflet (Devaux, 1991; Zachowski, 1993; Murate and Kobayashi, 2016). The enrichment of aminophospholipids in the cytosolic leaflet of the plasma membrane and at the surface of endocytic and secretory vesicles is required to keep these membranes in a fusion-competent state (Kinnunen and Holopainen, 2000).

Maintenance and regulation of membrane lipid asymmetry is governed by a set of enzymes known as lipid flippases. Among these, the type 4 subfamily of P-type adenosine triphosphatases (P4-ATPases) comprises flippases that transport lipids from the exoplasmic leaflet to the cytosolic leaflet of cellular membranes (Andersen et al., 2016; Shin and Takatsu, 2019). Most P4-ATPases are known to associate with an accessory β subunit known as cell division control protein 50 (CDC50), thus forming a heterodimeric complex (Bryde et al., 2010; van der Velden et al., 2010; Naito et al., 2015; Segawa et al., 2016). This association is required for both proper localization and activity of the P4-ATPase (Paulusma et al., 2008; Bryde et al., 2010; van der Velden et al., 2010; Naito et al., 2015; Segawa et al., 2016), but does not affect its substrate specificity (López-Marqués et al., 2010). In mammals, the CDC50 family consists of only three members: CDC50A, CDCD50B and CDC50C (also named TMEM30A, TMEM30B, TMEM30C, respectively). Since the mammalian genome encodes fourteen P4-ATPases and only three CDC50 proteins, a given CDC50 protein is likely to interact with multiple P4-ATPases.

In addition to these energy-dependent lipid flippases, mammalian cells contain lipid scramblases, such as transmembrane protein 16F (TMEM16F, also known as ANO6) and XK-related protein 8 (XKR8) (Suzuki et al., 2010; 2013; Hochreiter-Hufford et al., 2013; Park et al., 2016), that upon activation facilitate a rapid bidirectional movement of lipids between the two plasma membrane leaflets, dissipating the membrane lipid asymmetry. Regulated loss of lipid asymmetry at the plasma membrane, which results in the exposure of aminophospholipids at the cell surface, is important in numerous biological processes, including skeletal muscle development. A primary event in the development of skeletal muscle is the fusion of the plasma membranes of neighboring mononucleated myoblasts to form multinucleated myotubes, which finally bundle to form mature muscle fibers (Horsley and Pavlath, 2004). This process requires a transient exposure of PS in the outer leaflet of the plasma membrane of fusion-committed myoblasts at cell–cell contact sites (Sessions and Horwitz, 1983; van den Eijnde et al., 2001; Kašpar and Dvořák, 2008; Jeong and Conboy, 2011). Furthermore, recognition of PS by the BAI1 receptor (also known as ADGRB1) induces a signal for myoblast fusion during myoblast differentiation (Hochreiter-Hufford et al., 2013), and the interaction between stabilin-2 and exposed PS on the surface of healthy myoblasts promotes myotube formation (Park et al., 2016). Regulated cell-surface exposure of PS is also important for the regulation of PIEZO1, a mechanosensitive Ca^2+^ channel involved in the control of polarized membrane fusion (Tsuchiya et al., 2018). However, little is known about the transbilayer dynamics of other phospholipids across the plasma membranes of skeletal muscle cells and the involvement of lipid transporters in myoblast fusion.

In this study, we investigate the internalization of fluorescent phospholipids from the plasma membrane of mouse C2C12 myoblasts during both proliferation and the early phases of differentiation. Our results show that myoblasts exhibit an aminophospholipid flippase activity that relies on CDC50A-dependent P4-ATPases. The absence of CDC50A causes significant impairment of myotube formation following differentiation stimulation, whereas deletion of the P4-ATPase ATP11A affects aminophospholipid uptake without having a strong impact on cell fusion. These results demonstrate that cell fusion depends on CDC50A and may require more than one CDC50A-dependent P4-ATPase.

## RESULTS

### Changes of total lipid composition during C2C12 cell differentiation

**Fig. 1.**
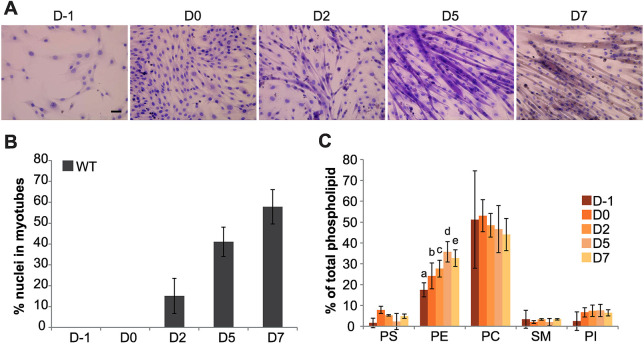
**Changes of lipid composition during C2C12 cell differentiation.** (A) Analysis of differentiation using multicolor Basic Fuchsin–Toluidine Blue staining. Representative microscopy images taken during proliferation (D−1) and differentiation at days 0 (D0), 2 (D2), 5 (D5) and 7 (D7) are shown. Images are representative of three independent experiments. Scale bar: 50 μm. (B) Percentage fusion of wild-type (WT) C2C12 cells, calculated by dividing the number of nuclei within multinucleated myofibers by the total number of nuclei in the sample. Data represent the mean±s.d. of three independent experiments. (C) Total phospholipid composition of C2C12 cells. Data are expressed as the percentage of total phospholipids and represent the mean±s.d. of three independent experiments. Error bars with letters represent significant differences between groups (two-way ANOVA and Tukey's HSD test; *P*<0.01). PC, phosphatidylcholine; PE, phosphatidylethanolamine; PI, phosphatidylinositol; PS, phosphatidylserine; SM, sphingomyelin.

To elucidate the phospholipid dynamics during C2C12 cell differentiation, we first evaluated the differentiation of myoblasts into elongated multinucleated myotubes using Basic Fuchsin–Toluidine Blue staining (Fig. 1). C2C12 cells were seeded in growth medium on day −1 (D−1) to achieve ∼100% confluency at day 0 (D0), followed by culturing in differentiation medium for 7 days (D1–D7). Under these conditions, myotube formation began at day 2 and increased progressively (Fig. 1A). After 7 days of differentiation, ∼58% of mononucleated myoblasts were fused into multinucleated myotubes (Fig. 1B). Analysis of the total phospholipid composition in proliferating and differentiated C2C12 cells using thin layer chromatography and lipid phosphorus determination showed that the relative amounts of PE increased gradually during myoblast differentiation and fusion (from 17.5%±3.4 at D−1 to 32.7%±4.0 at D7; mean±s.d.) (Fig. 1C).

### C2C12 cells display aminophospholipid flippase activity at the plasma membrane

**Fig. 2.**
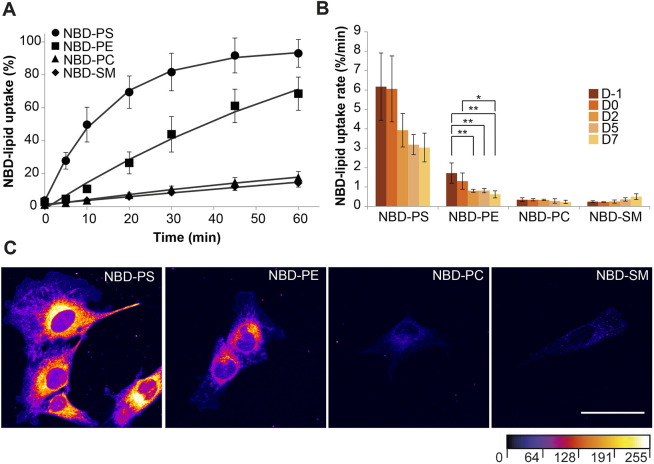
**Internalization of NBD-labeled lipids from the plasma membrane of proliferating and differentiating C2C12 cells.** (A) Timecourse of NBD-labeled lipid internalization in proliferating C2C12 myoblasts measured by flow cytometry using back-exchange to albumin. Plotted lines represent the best fit to a single-exponential curve. Data are mean±s.d. from three independent experiments. (B) NBD-labeled lipid internalization rates in proliferating (D−1) and differentiating C2C12 cells at days 0 (D0), 2 (D2), 5 (D5) and 7 (D7), calculated by fitting the data to a single-exponential equation. Data are mean±s.d. from three independent experiments (two-way ANOVA and Tukey’s HSD test; *P<0.05; **P<0.01). (C) Representative confocal images of proliferating C2C12 cells labeled with the indicated NBD-labeled lipids for 60 min and subjected to back-exchange with albumin. Images are color-coded with the FIRE look-up table (ImageJ) to highlight intensity variations. Images are representative of three independent experiments. Scale bar: 50 μm.

To study the activity of lipid transporters in the plasma membrane of C2C12 cells during differentiation, we next examined the uptake of nitrobenzoxadiazole (NBD)-labeled lipids using flow cytometry. Such fluorescent lipid analogs are well suited to qualitatively reflect the transmembrane dynamics and distribution of their endogenous counterparts. Experiments were performed at 20°C to suppress both endocytosis and the metabolic conversion of the NBD-labeled lipids (integrity for NBD–PE >80%, for all other lipids >95% after 60 min of incubation). Under these conditions, proliferating C2C12 myoblasts efficiently internalized NBD–PS and NBD–PE, with transport rates at least 6- and 4-fold higher, respectively, than those of NBD-labeled phosphatidylcholine (PC) and sphingomyelin (SM) (Fig. 2A,B). Consistent with these results, fluorescence microscopy revealed an intensive intracellular labeling of membranes throughout the cells with NBD–PS and NBD–PE (Fig. 2C). In contrast, NBD–PC and NBD–SM were hardly internalized and were predominately detected in intracellular punctate structures. During myoblast differentiation (D0 to D7), the transport rates of NBD–PS and NBD–PE dropped to 75% and 43%, respectively, as compared to those in proliferating myoblasts (Fig. 2B). These observations were further confirmed using L6 rat skeletal muscle cells incubated with spin-labeled lipids (Fig. S1). Collectively, these data are consistent with the presence of an active inward aminophospholipid transport activity at the plasma membrane of C2C12 cells, which is downregulated during the differentiation of myoblasts into myotubes.

### Expression of P4-ATPases and β subunits during C2C12 cell proliferation and differentiation

**Fig. 3.**
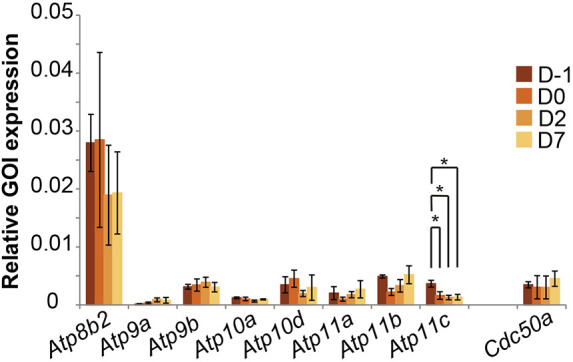
**Expression of P4-ATPases and β subunits in C2C12 cells.** Analysis of mRNA expression for P4-ATPases and CDC50 family members in proliferating (D−1) and differentiating (D0–D7) C2C12 cells. Ct values of genes of interest (GOI) were normalized to the housekeeping gene *Gapdh*. Data represent the mean±s.d. of three independent experiments (two-way ANOVA and Tukey's HSD test; *P<0.05).

The prime candidates for inward lipid transporters are members of the P4-ATPase family in complex with their CDC50 subunit. We therefore carried out reverse transcription-quantitative PCR (RT-qPCR) experiments to study the expression of all 15 mouse P4-ATPases and three CDC50 proteins in C2C12 cells. In proliferating myoblasts, this analysis revealed the expression of several P4-ATPase-encoding genes, including *Atp8b2*, *Atp9b*, *Atp10a*, *Atp10d*, *Atp11a*, *Atp11b* and *Atp11c*, together with one single β subunit protein, *Cdc50a* (Fig. 3). This indicates that C2C12 cells contain CDC50A as the only P4-ATPase interaction partner, in line with previous results (Tsuchiya et al., 2018). Expression levels did not change under myogenic differentiation conditions, except for the P4-ATPase *Atp11c*, which was significantly downregulated throughout the culture period.

### CDC50A is required for aminophospholipid internalization and normal cell fusion in C2C12 cells

All P4-ATPases expressed in C2C12 cells, with the exception of *Atp9b*, have been reported to require a β subunit of the CDC50 protein family for endoplasmic reticulum export and catalytic activity of the enzyme complex (Paulusma et al., 2008; Bryde et al., 2010; van der Velden et al., 2010; Takatsu et al., 2011; Naito et al., 2015; Segawa et al., 2016). Since C2C12 cells only express the *Cdc50a* β subunit, we decided to first study the potential implications of CDC50A on lipid transport across the plasma membrane. To this end, CDC50A-deficient myoblasts were generated using the CRISPR/Cas9 system. To rule out potential unspecific Cas9-mediated DNA damage, we chose three independent cell clones that were tested homozygous for CRISPR-induced recombination for subsequent experiments. In all three cell clones, *Cdc50a* transcripts were absent as assessed by RT-qPCR analysis (Fig. S2A).

**Fig. 4.**
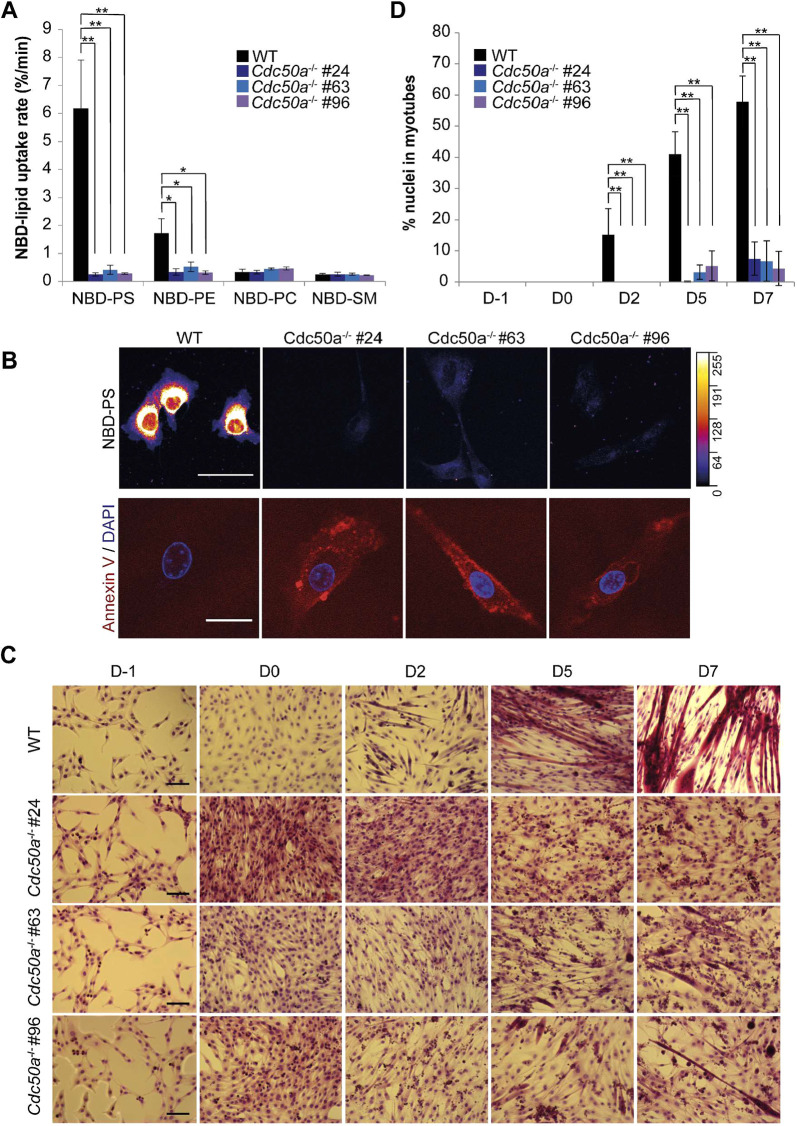
**CDC50A-deficient C2C12 cells display defects in aminophospholipid uptake and cell fusion.** (A) NBD-labeled lipid internalization rates in proliferating wild-type (WT) and CDC50A-deficient cells (clones #24, #63 and #96). Cells were labeled with the indicated NBD-labeled lipids at 20°C for 60 min and analyzed as described in the Materials and Methods. (B) Representative confocal images of proliferating C2C12 cells labeled with NBD–PS for 60 min (upper panel) or Alexa Fluor 568-conjugated annexin V (lower panel, red). DAPI (lower panel, blue) was used to identify nuclei. The ImageJ FIRE look-up table was used to highlight NBD–PS intensity variations. Images are representative of three independent experiments. Scale bars: 50 μm. (C) Analysis of differentiation by multicolor Basic Fuchsin–Toluidine Blue staining. Representative microscopy images of wild-type and CDC50A-deficient cells (clones #24, #63 and #96) during proliferation (D−1) and differentiation at days 0 (D0), 2 (D2), 5 (D5) and 7 (D7). Images are representative of three independent experiments. The experiment depicted was performed in parallel with the experiment shown in Fig. 6B, using the same WT samples. The WT images shown here are also shown in Fig. 6B. Scale bars: 100 μm. (D) Calculation of fusion indices for the indicated cell lines, showing that *Cdc50a^−/−^* myoblasts are severely deficient in their fusion capacity, with the majority of cells containing single nuclei. Data in panel A and D represent the mean±s.d. of two independent experiments (two-way ANOVA and Tukey’s HSD test; *P<0.05, **P<0.01).

Analysis of NBD-labeled lipid uptake revealed a complete loss of aminophospholipid uptake for all three CDC50A-deficient cell clones (Fig. 4A; Fig. 4B, upper panel; Fig. S3). To visualize the effect of *Cdc50a* deletion on lipid asymmetry in the plasma membrane, we stained the cells with Alexa Fluor 568-conjugated annexin V, a protein that binds to PS and PE in a Ca^2+^-dependent manner (Swairjo et al., 1995; Stuart et al., 1998), as confirmed using giant vesicles with defined lipid compositions (Fig. S4). As shown in Fig. 4B (lower panel), all three CDC50A-deficient cell clones, but not proliferating wild-type cells, stained positive for annexin V, indicating increased surface exposure of aminophospholipids upon the loss of *Cdc50a*.

**Fig. 5.**
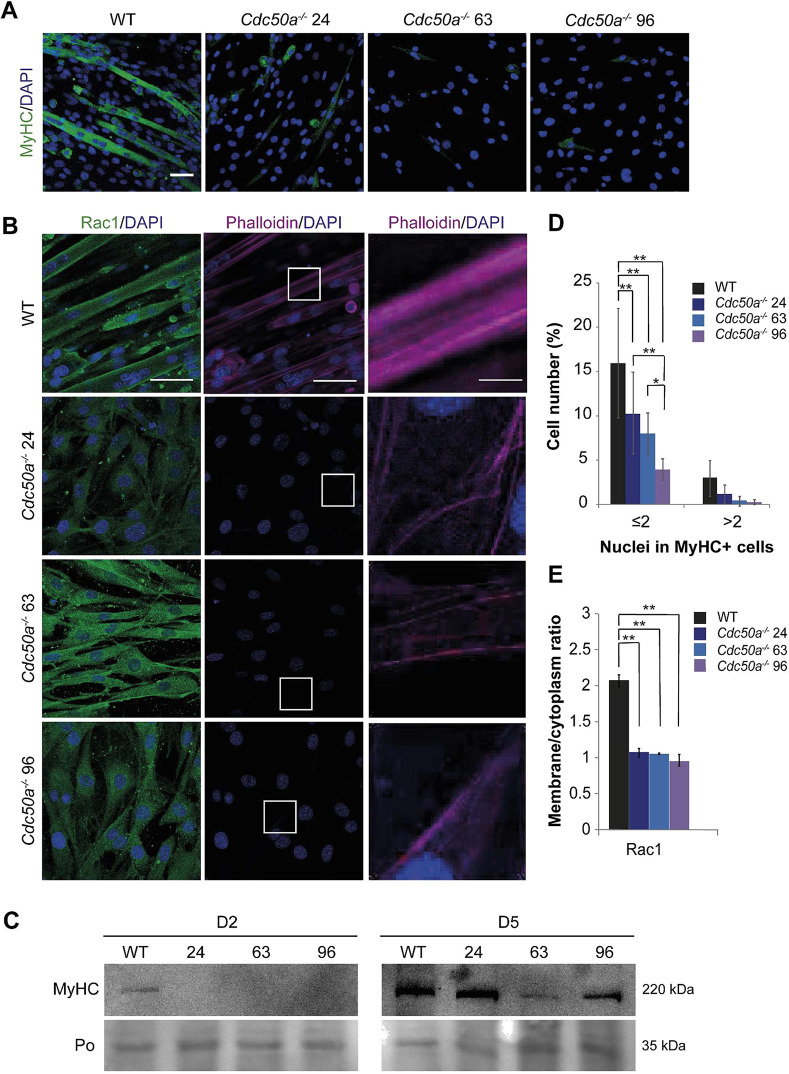
**Loss of CDC50A affects actin remodeling and RAC1 plasma membrane localization.** (A) Immunofluorescence of MyHC (green) and nuclei (DAPI, blue) in wild-type (WT) and CDC50A-deficient cells (clones #24, #63 and #96) at day 2 of differentiation. Scale bar: 50 μm. (B) Immunofluorescence of RAC1 (left panel, green) and F-actin (middle panel, phalloidin, red) in wild-type and CDC50A-deficient cells (clones #24, #63 and #96) at day 2 of differentiation. The nuclei are stained with DAPI (blue). Right panels show zoomed-in images of regions indicated by white boxes in the middle panels, adjusted with different tonal values, brightness and contrast to enhance the low signal intensities. Scale bars: 50 µm (left and middle panels), 10 μm (right panel). (C) Western blot of total cell extracts from the indicated wild-type and CDC50A-deficient cells at day 2 (D2) and day 5 (D5) of differentiation demonstrates the relative level of MyHC. Ponceau (Po) staining was performed to confirm equivalent protein loading. Blots are representative of two independent experiments. (D) Percentage of MyHC-positive cells at day 2 of differentiation grouped by number of nuclei. (E) Quantification of plasma membrane:cytoplasm RAC1 fluorescence for the experiment shown in B. Data in panels D and E represent the mean±s.d. of two independent experiments (two-way ANOVA and Tukey’s HSD test; *P<0.05; **P<0.01).

Microscopic inspection showed that the three CDC50A-deficient cell clones grew well and had a triangular morphology similar to that of wild-type cells under proliferating growth conditions (Fig. 4C; D−1). However, when cultured under conditions that promote differentiation, CDC50A-deficient cells at low passage numbers (<20) exhibited severe defects in adopting a bipolar shape and forming multinucleated myotubes (Fig. 4C; D0–D7). The fusion index of wild-type cultures was ∼58% after 7 days of differentiation, with an average of 6.9±2.1 nuclei per myotube (mean±s.d.; Fig. 4D). In contrast, by day 7 less than 8% of CDC50A-deficient cells contained 2 or 3 nuclei, with an average of 1.8±1.9 nuclei per myotube for clone 24, 2.5±1.9 for clone 63 and 3.1±3.2 for clone 96. Notably, at higher passage numbers (>20), we observed that a larger number of CDC50A-deficient cells did fuse, but they formed large, morphologically abnormal multinucleated syncytia (Fig. S5A,C). Similar results were obtained for cells transduced with lentivirus to deliver short hairpin RNA (shRNA) directed against *Cdc50a* (Figs S5E,F and S6), excluding the possibility that the phenotypes observed for CDC50A-deficient cells were caused by off-target effects. The reduced ability of CDC50A-deficient cells to form multinucleated myotubes was paralleled by an interference with the biochemical differentiation, as revealed by examining the expression of the late-stage differentiation marker myosin heavy chain II (MyHC, specifically the light meromyosin portion). Analysis by immunofluorescence after 2 days in differentiation medium showed that CDC50A-deficient cells contained fewer MyHC-positive cells than did equivalent control cultures (Fig. 5A,D and Fig. S6D,E). Western blot analysis confirmed the lower expression of MyHC in CDC50A-deficient cells as compared to levels in wild-type cells (Fig. 5C). Thus, loss of CDC50A not only affects myoblasts in their competence to form multinucleated myotubes but also in their differentiation capacity.

### CDC50A deficiency affects actin remodeling and RAC1 plasma membrane localization

Adoption of bipolar shape and fusion of myoblasts requires reorganization of the actin cytoskeleton, which is regulated by small guanosine triphosphatases (GTPases) of the Rho family, such as RAC1 (Peckham, 2008; Duan and Gallagher, 2009; Vasyutinaa et al., 2009). Thus, we examined RAC1 distribution and actin arrangement in control and CDC50A-deficient cells. Phalloidin staining of control cultures at day 2 of differentiation revealed long filament bundles of F-actin localizing to the cell periphery and spanning the entire length of the cells (i.e. spindle shaped, mononuclear myoblasts and multinucleate myotubes). In contrast, CDC50A-deficient cells showed reduced phalloidin staining with several randomly orientated filaments in the cytoplasm (Fig. 5B; Fig. S6D). Furthermore, CDC50A-deficient cells displayed a reduced plasma membrane association of RAC1, as revealed by confocal fluorescence immunohistochemistry (Fig. 5B,E). Taken together, these observations suggest that loss of CDCD50A affects actin remodeling and RAC1 recruitment to the plasma membrane.

### Loss of ATP11A affects aminophospholipid uptake but not cell fusion in C2C12 cells

**Fig. 6.**
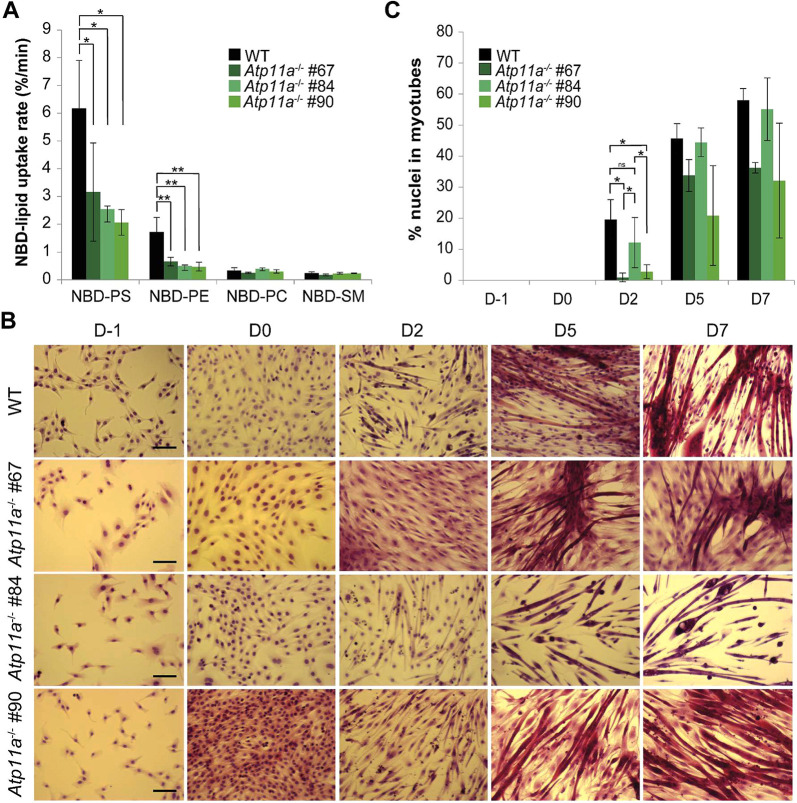
**Reduced aminophospholipid uptake in ATP11A-deficient cells does not impair cell fusion.** (A) NBD-labeled lipid internalization rates in proliferating wild-type (WT) and ATP11A-deficient cells (clones #67, #84 and #90). Cells were labeled with the indicated NBD-labeled lipids at 20°C for 60 min and analyzed as described in the Materials and Methods. (B) Analysis of differentiation by multicolor Basic Fuchsin–Toluidine Blue staining. Representative microscopy images of wild-type and three low passage ATP11A-deficient cell lines during proliferation (D−1) and differentiation (D0, D2, D5 and D7). Images are representative of three independent experiments. The experiment depicted was performed in parallel with the experiment shown in Fig. 4C, using the same WT samples. The WT images shown here are also shown in Fig. 4C. Scale bars: 100 μm. (C) Fusion index calculated at the indicated days of differentiation. Data in panel A and C represent the mean±s.d. of two independent experiments (two-way ANOVA and Tukey’s HSD test; *P<0.05; **P<0.01; ns, not significant).

Among the P4-ATPases expressed in C2C12 cells, ATP11A represents an aminophospholipid transporter at the plasma membrane previously found to be important in myoblast fusion (Tsuchiya et al., 2018). We therefore next generated ATP11A-deficient cells using the CRISPR/Cas9 system and isolated three independent clones for further analysis. In all three cell clones, *Atp11a* transcripts were absent, as assessed by RT-qPCR analysis (Fig. S2B). Deletion of *Atp11a* led to a marked decline in the uptake rate of NBD–PS and NBD–PE without affecting the slow internalization of NBD–PC and NBD–SM (Fig. 6A). Under proliferating growth conditions, ATP11A-deficient cells showed a similar morphology to wild-type cells (Fig. 6B). Under differentiating conditions, both low passage (<20) wild-type and ATP11A-deficient cells aligned with each other and formed long parallel bundles of multinucleated myotubes, showing no defects in cell fusion. In contrast to previous findings (Tsuchiya et al., 2018), morphologically abnormal multinucleated syncytia for ATP11A-deficient cells were observed for less than half of the cells at day 7 (i.e. 58 out of 352 myotubes for clone 67, 56 out of 222 myotubes for clone 84, 115 out of 287 myotubes for clone 90). Additionally, we noted a slightly delayed onset of cell fusion for two ATP11A-deficient cell clones, as revealed by fusion index determination (Fig. 6C, clones 67 and 90). At high passage numbers (>20), however, ATP11A-deficient cells displayed reduced cell fusion into morphologically abnormal multinucleated syncytia (Fig. S5B,D).

## DISCUSSION

Past work has indicated that formation of multinucleated myotubes is accompanied by changes in membrane lipid composition (Wakelam, 1985; Yin et al., 2009), in lipid domain organization of the plasma membrane (Mukai et al., 2009) and in the transbilayer lipid distribution across the plasma membrane (van den Eijnde et al., 2001; Jeong and Conboy, 2011; Hochreiter-Hufford et al., 2013; Park et al., 2016). The findings presented in this work further emphasize the importance of membrane lipid composition and transbilayer lipid dynamics in myoblast fusion.

Lipid analysis by thin layer chromatography revealed a significant increase of the cellular PE levels during myoblast differentiation (Fig. 1). This increase correlates with recent results showing a marked increase in the cellular content of PE during osteoclast differentiation (Irie et al., 2017). Similar to myoblasts, osteoclasts undergo fusion to form multinucleated cells. During fusion, membranes have to come into close contact, which requires removal of bound water from the headgroup of the lipid molecules followed by local destabilization of the lipid bilayer. Like PS, PE meets these fusogenic properties, because the headgroup of this lipid is less hydrated than that of other lipids and supports the formation of non-bilayer structures during cell fusion (Chernomordik and Kozlov, 2003; Kozlov, 2007). Further studies are needed to ascertain whether the increase in the cellular PE levels is accompanied by a concomitant increase in plasma membrane PE levels.

Incubation of cells with fluorescent phospholipid analogs (PC, PE, PS and SM) served as an approach to analyze lipid transport activities across the plasma membrane of C2C12 myoblasts during proliferation and differentiation. For this to be a valid approach, both internalization by endocytosis and metabolic conversion of the lipid analogs should be negligible. Under conditions that minimize these factors, C2C12 myoblasts hardly take up NBD–PC and NBD–SM but efficiently internalize NBD–PS and NBD–PE. These data are consistent with the presence of an active aminophospholipid flippase activity at the plasma membrane of proliferating C2C12 cells. During differentiation of myoblasts into myotubes, internalization of NBD–PS and NBD–PE (but not of NBD–PC and NBD–SM) dropped, indicating a downregulation of the aminophospholipid-specific flippase activity. Similar results were obtained for L6 rat skeletal muscle cells (Fig. S1), suggesting that these findings are species independent. Recent studies have in addition indicated an important role of lipid scramblases (i.e. ANO5) in the regulation of membrane lipid organization during myoblast fusion (Kim et al., 2017; Whitlock et al., 2018). Thus, downregulation of the aminophospholipid flippase activity accompanied by a local increase in phospholipid scramblase activity can explain the previously reported transient exposure of PS in the outer leaflet of the plasma membrane of fusion-committed myoblasts at cell–cell contact sites during myoblast differentiation (van den Eijnde et al., 2001; Kašpar and Dvořák, 2008; Jeong and Conboy, 2011).

A major group of lipid flippases comprises P4-ATPases in complex with their β subunits of the CDC50 protein family. Using RT-qPCR, we found that C2C12 myoblasts express seven P4-ATPases (ATP8B2, ATP9B, ATP10A, ATP10D, ATP11A, ATP11B and ATP11C) along with the single β subunit CDC50A. Among the P4-ATPases expressed in C2C12 cells, ATP11A, ATP11B and ATP11C are classified as aminophospholipid transporters that require CDC50A as a partner and might have redundant roles (Takatsu et al., 2014; Segawa et al., 2016; Wang et al., 2018), making it difficult to study their functions. We therefore first focused on the β subunit of these P4-ATPases. Both deletion and shRNA-mediated depletion of CDC50A abolished the rapid internalization of aminophospholipids across the plasma membrane of C2C12 myoblasts, indicating that CDC50A-dependent P4-ATPases facilitate the transport of natural PS and PE from the outer to the inner leaflet of the myoblast plasma membrane. In support of this notion, loss of CDC50A resulted in an increased cell surface exposure of natural PS and PE, as evidenced by labeling with annexin V. These results are in line with a direct role of the CDC50A-dependent P4-ATPases in flipping natural PS and PE to the cytosolic plasma membrane leaflet to generate and maintain transbilayer lipid asymmetry. C2C12 cells deleted for the cell surface-localized P4-ATPase ATP11A displayed only a partial reduction of aminophospholipid uptake, suggesting that other P4-ATPases can functionally compensate for the loss of ATP11A. Prime candidates for these aminophospholipid flippases are ATP11B and ATP11C, although ATP11B has been primarily localized to intracellular membranes (Okamoto et al., 2020). Further studies are required to determine the subcellular localization and the contribution of these P4-ATPases in muscle cells.

Recent work has indicated that the phospholipid flippase complex of ATP11A and CDC50A acts as a molecular switch for the activation of the mechanosensitive PIEZO1 channel, which governs proper morphogenesis during myotube formation (Tsuchiya et al., 2018). In this previous study, the authors showed that C2C12 cells lacking either ATP11A or CDC50A undergo excessive fusion to large, morphologically abnormal multinucleated syncytia. In our experiments, CDC50A-deficient C2C12 cells were viable and grew well, confirming that CDC50A-dependent P4-ATPases are not essential for myoblast proliferation. Under differentiation conditions, however, CDC50A-deficient cells at low passage number were severely impaired in fusing to multinucleated myotubes. Lack of ATP11A, on the other hand, merely slowed down myotube formation, but only a low percentage of the myotubes displayed aberrantly enlarged syncytia under our experimental conditions. These discrepancies between the data are likely caused by different variables in the experimental procedure, most importantly the passage number of the cells. We observed that, in contrast to low passage CDC50A-knockout cells, high passage CDC50A-deficient cells displayed an increasing fusion ability and formed numerous abnormal multinucleated syncytia, as reported previously (Tsuchiya et al., 2018). These observations suggest that at higher passage number the knockout cells try to exploit compensatory mechanisms to correct for the fusion problem. Similar passage number-dependent observations were found in ATP11A-deficient cells. Taken together, our findings indicate that the regulation of myoblast fusion is more complex than previously anticipated and may involve both CDC50A-dependent (including different members of the P4-ATPase family) and CDC50A-independent pathways. The detailed role of P4-ATPases in myoblast fusion remains to be determined.

A critical question is how the deficiency of CDC50A-dependent P4-ATPases leads to impaired myoblast fusion. A crucial event in membrane fusion is the rearrangement of the actin cytoskeleton, which is tightly regulated by Rho GTPases, including CDC42 and RAC1, and their regulatory proteins (Peckham, 2008; Duan and Gallagher, 2009; Vasyutinaa et al., 2009). Studies in *Saccharomyces cerevisiae* have revealed that P4-ATPase-mediated lipid translocation at the plasma membrane is essential for dissociation of Cdc42p from the cell cortex, leading to a blockage of actin polymerization and triggering of the growth switch of the growing bud tip of the daughter cell (Saito et al., 2007; Das et al., 2012). Among mammalian P4-ATPases, ATP9A and ATP8B1 are involved in the recruitment and clustering of Rho GTPases at the plasma membrane (Kato et al., 2013; Bruurs et al., 2015). Results presented in this study show that CDC50A deficiency in myoblasts resulted in a loss of aminophospholipid transport and asymmetry that diminished the association of the small GTPase RAC1 with the plasma membrane. These changes were accompanied by defects in the rearrangements of the actin cytoskeleton at the plasma membrane, in line with previous observations (Tsuchiya et al., 2018). Conceivably, lipid translocation by P4-ATPase–CDC50A protein complexes generates a local membrane environment suitable for the recruitment and clustering of the protein machinery that controls remodeling of the cytoskeleton and cell fusion at the plasma membrane. In addition to their function as lipid flippases, P4-ATPase–CDC50A protein complexes may also provide a molecular scaffold in the membrane to recruit structural components or modulators of the actin cytoskeleton. For example, the yeast P4-ATPase Drs2p has been found to directly interact with cytosolic proteins such as guanine-nucleotide-exchange factors and small GTPases that are crucial for the recruitment of coat proteins during membrane budding (Chantalat et al., 2004; Furuta et al., 2007; Tsai et al., 2013). Thus, CDC50A in complex with P4-ATPases may help recruitment of Rho GTPases at the myoblast plasma membrane. Given that Rho GTPases are also involved in controlling nuclear signaling, gene expression and cell differentiation (Takano et al., 1998; Meriane et al., 2000; Travaglione et al., 2005), improper recruitment of these regulators to the plasma membrane may explain the impaired biochemical differentiation observed for CDC50A-deficient cells.

## MATERIALS AND METHODS

### Chemicals

Culture media and reagents were purchased from Sigma-Aldrich (Brøndby, Denmark), if not stated otherwise (Table S1). Growth medium (GM) consisted of high-glucose Dulbecco's Modified Eagle's Medium (DMEM) supplemented with 20% heat-inactivated fetal calf serum. Differentiation medium (DM) consisted of low-glucose DMEM with 2% heat-inactivated horse serum. Hanks’ balanced salt solution (HBSS) contained 137 mM NaCl, 5.4 mM KCl, 0.3 mM Na_2_HPO_4_, 0.4 mM KH_2_PO_4_, 5 mM glucose and 4 mM NaHCO_3_. Tyrode's Balanced Salt Solution (TBSS) contained 136 mM NaCl, 2.6 mM KCl, 1.8 mM CaCl_2_, 1 mM MgCl_2_, 0.36 mM NaH_2_PO_4_, 5.56 mM D-glucose and 5 mM 4-(2-hydroxyethyl)-1-piperazineethanesulfonic acid, pH 7.4. The 7-nitrobenz-2-oxa-1,3-diazol-4-yl (NBD) lipid derivatives palmitoyl-(NBD-hexanoyl)-phosphatidylcholine (NBD–PC), palmitoyl-(NBD-hexanoyl)-phosphatidylethanolamine (NBD–PE), palmitoyl-(NBD-hexanoyl)-phosphatidylserine (NBD–PS) and 6-NBD-hexanoyl-sphingosine-1-phosphocholine (NBD-sphingomyelin; NBD–SM) were purchased from Avanti Polar Lipids (Birmingham, AL, USA). All spin-labeled analogs were synthesized as described by Morrot et al. (1989). FM4-64 was from Invitrogen, Molecular Probes (Eugene, OR, USA); Alexa Fluor 568-conjugated annexin V was from Roche Molecular Biologicals (Mannheim, Germany).

### Cell culture

Mouse C2C12 myoblasts (ATCC CRL-1772; Blau et al., 1985) and rat L6 myoblasts (ATCC CRL-1458; Yaffe, 1968) were maintained in uncoated standard tissue culture plastic flasks in a humidified incubator at 5% CO_2_ and 37°C in GM. At 70% confluency, cells were trypsinized (0.125% trypsin, 0.02% EDTA in HBSS) and split at a 1:3 ratio. Myotube formation was induced by replacing GM with DM. DM was changed every day. To knockout the *Cdc50a* or *Atp11a* genes, C2C12 cells were transfected with a combination (1:1) of CRISPR-Cas9 and homology-directed repair (HDR) reporter vectors (Santa Cruz Biotechnology, Heidelberg, Germany) and selected with puromycin (1 μg ml^−1^). Biallelic integration of the HDR cassette to genomic loci was confirmed by PCR using the primers listed in Table S2.

### Knockdown of CDC50A using shRNA

CDC50A-knockdown cells were generated by lentiviral transduction of C2C12 cells. Briefly, C2C12 cells were incubated with virus-containing supernatants in GM supplemented with 10 µg/ml diethylaminoethyl-dextran (Pharmacia Fine Chemical, Uppsala, Sweden) for 4 h, after which the medium was refreshed. Two days post-transduction, cells were selected with 2 µg/ml puromycin. A validated short hairpin RNA (shRNA) sequence targeting the murine *Cdc50a* (*Tmem30a*) mRNA (5′-CGTAAGTTGTATCGTCTCATA-3′; TRCN0000087891) and a non-targeting shRNA (SHC002; 5′-CAACAAGATGAAGAGCACCAA-3′; TRC1.5 control vector) were obtained through the MISSION shRNA library (Sigma-Aldrich).

### Quantification of myotube fusion

For analysis of myotube fusion, cells were washed in HBSS and then fixed in 70% ethanol for 5 min. After ethanol removal, a multiple stain containing 30 mM Toluidine Blue and 8 mM Basic Fuchsin (Merck, Søborg, Denmark) in 30% ethanol was added (McColl et al., 2016). After 5 min, cells were washed ten times with distilled water, and five to ten fields of view were randomly imaged using a 20× objective mounted on an inverted light microscope (Leica DMI 4000B) equipped with a CCD camera (Leica DFC310FX). For each experimental condition and time point, the number of nuclei in each myotube (≥3 nuclei) and the total number of nuclei in cells were counted in at least three fields of view of at least two independent culture flasks, and the fusion index (in %) was calculated as (number of nuclei in myotubes/total number of nuclei)×100. The analysis included >600 nuclei, except for day −1 (>100 nuclei) due to the low cell number at this culture stage. Average fusion indices and s.d. were calculated using Microsoft Excel (Microsoft Corporation, Redmond, USA).

### RT-qPCR

To differentiate between cDNA and genomic DNA, primers were designed at distinct sites of the exon–exon boundaries (Table S3). All primers were synthesized by Integrated DNA Technologies (Leuven, Belgium). Total cellular RNA was extracted using the RNA isolation NucleoSpin kit (Macherey-Nagel, Düren, Germany) according to the manufacturer's instructions. RT-PCR was performed using QuantiTect SYBR Green RT-PCR kit (Qiagen, Hilden, Germany), reaction capillaries (Roche Diagnostics, Mannheim, Germany) and a Light Cycler 1.5 (Roche Diagnostics). The amplification program started with the cDNA synthesis by a reverse transcription step at 50°C for 20 min, followed by pre-denaturation at 95°C for 15 min and 22–45 cycles of amplification (denaturation for 10 s at 95°C, annealing for 20 s at 59°C and elongation at 72°C for 40 s). For each reaction, the cycle threshold (Ct) was determined using the 2nd derivative method of the LightCycler 480 Software, release 1.5 (Roche). The relative gene expression levels were expressed as the difference in Ct values of the target gene and *Gapdh*. For knockout and wild type comparison analysis, knockout Ct values were calculated relative to *Gapdh* and wild-type cells according to the 2^−ΔΔCt^ method (Schmittgen and Livak, 2008).

### Lipid uptake assay

Appropriate amounts of NBD-labeled lipids (dissolved in chloroform/methanol) were transferred to a glass tube, dried under nitrogen and dissolved into 5 μl DMSO. For labeling of cells in suspension, cells were harvested by trypsination and resuspended in TBSS. To block the catabolism of NBD-lipids, cells were pre-incubated for 10 min at 20°C with 1 mM of phenylmethanesulphonyl fluoride (from a 200 mM ethanol stock) and 5.8 μM of 3-(4-octadecyl)-benzoylacrylic acid (Tocris, Wiesbaden-Nordenstadt, Germany; from a 5.8 mM ethanol stock). The uptake assay was set up in glass tubes at 20°C by mixing 2 ml of 10^7^ cells in suspension with the NBD-labeled lipids. At the indicated time points, two 100 μl aliquots were removed. One aliquot was stored on ice to determine the total fluorescence associated with the cells. The other aliquot was mixed with 5% (w/v) fatty acid-free bovine serum albumin (BSA) in TBSS, to extract NBD-labeled lipids from the cell surface. The cell aliquots were analyzed within one hour by flow cytometry. For microscopy, cells grown on 35 mm glass-bottom Petri dishes (MatTeK Co., Ashland, MA) were labeled with NBD-labeled lipids as described above and incubated for 60 min at 20°C. Subsequently, cells were washed twice with TBSS supplemented with BSA (5%, w/v) for 2 min, and then maintained in TBSS for analysis using fluorescence microscopy. Transport assays with spin-labeled lipids were performed essentially as described previously (Pomorski et al., 1996).

### Flow cytometry and lipid uptake analysis

Flow cytometry analysis was performed on a CyFlow SL (Partec, Münster, Germany) equipped with an argon laser (488 nm). Just before analysis, 1 μl of 1 mg ml^−1^ propidium iodide in H_2_O was added to the cell suspension. Ten thousand cells were analyzed at room temperature without gating during the acquisition. A histogram of the red fluorescence (propidium iodide; LP 630 nm) was used to set the gate that excluded dead cells from the analysis. Green fluorescence (NBD; BP 527/30) of living cells was plotted on a histogram. Data was analyzed using the FlowJo software (Tree star, Ashland, USA), and the geometric mean fluorescence of each sample was determined. The percentage of uptake (*U*) for each NBD-labeled lipid was calculated as *U*=(*F*_BSA_/*F*_buffer_)×100, where *F*_BSA_ is the geometric mean fluorescence of the BSA-treated cells and *F*_buffer_ is the geometric mean fluorescence of control cells not treated with BSA.

### Annexin V–Alexa Fluor 568 binding assay

Cells grown on 35 mm glass-bottom MatTeK Petri dishes (MatTek Corporation, Ashland, MA, USA) were incubated for 20 min on ice with 5 µl Alexa Fluor 568-conjugated annexin V, washed twice with TBSS for 2 min, and subjected to fluorescence microscopy. To study annexin V binding to lipid membranes, giant vesicles were prepared as described previously (Weingärtner et al., 2012), diluted 1:2 in Ca^2+^-free or Ca^2+^-containing binding buffer supplemented with 1 µl Alexa Fluor 568-conjugated annexin V and incubated for 10 min before fluorescence microscopy.

### Immunofluorescence and immunoblotting

For immunofluorescence analysis, cells were cultured in 35 mm imaging dishes with an ibidi polymer coverslip bottom (ibidiTreat, Martinsfeld, Germany). Before staining, cells were washed with Dulbecco's Phosphate Buffered Saline (DPBS), fixed for 20 min with 4% paraformaldehyde at room temperature and permeabilized with 0.5% Triton X-100 for 15 min. Cells were blocked with 10% goat serum in DPBS containing 0.01% Triton X-100 and then stained with the indicated antibodies. Sister cultures in plastic flasks were lysed, and 13 μg of cleared cell extracts were examined by western blot analysis. As primary antibodies, Alexa Fluor 488-conjugated anti-MyHC monoclonal antibody (#53-6503-82, 5 µg ml^−1^, MF20; eBioscience, Thermo Fisher Scientific, Waltham, MA, USA) and anti-RAC1 antibody clone 23A8 (#05-389, 5 µg ml^−1^; Sigma-Aldrich) were used. As secondary antibodies, Alexa Fluor 488-conjugated F(ab’)2-goat anti-mouse IgG antibodies (A-24920, 4 µg ml^−1^; Thermo Fisher Scientific) were used for RAC1 detection. The actin cytoskeleton was labeled with phalloidin–TRITC (P1951, 50 µg ml^−1^; Sigma-Aldrich). Cell nuclei were stained with DAPI (Sigma-Aldrich; 0.5 µg ml^−1^).

### Fluorescence microscopy and imaging analysis

Fluorescence microscopy and image acquisition were carried out using a Leica TCS SP8 confocal laser scanning microscope (Leitz, Wetzlar, Germany) equipped with a 63×/1.20 water objective. Images were acquired using a 400 Hz unidirectional scanner, a pixel size of 2.77×2.77 μm, a pinhole of 111.5 μm (1 AU) and a frame averaging of four. The excitation and emission wavelengths used for imaging were as follows: NBD, 484/490–778 nm (excitation/emission); Alexa Fluor 488, 488/500–550 nm; Alexa Fluor 568, 576/600–630 nm; phalloidin–TRITC, 540–545/570–573 nm. The transmitted light (brightfield) images were recorded for each image. Images were scanned using the same conditions of pinhole, gain, laser power (20%) and detector offset in each experiment. At least five different images from random fields of view were captured and processed using the LasX (Leica microsystems) and ImageJ software (National Institutes of Health, Bethesda, MA). To study the distribution of RAC1 protein, small regions of interest were drawn over maximum projection images to define the plasma membrane and the cytoplasm, excluding the nucleus. The ratio of plasma membrane:cytoplasm intensity was calculated to normalize the intensity of the plasma membrane to the level of expression in each single cell using ImageJ software.

### Lipid analysis

Total cellular lipids were extracted by the method of Bligh and Dyer (Bligh and Dyer, 1959) and separated by thin layer chromatography. For phospholipid analysis, the plates were first developed in chloroform, methanol and 30% aqueous ammonium hydroxide (65:35:5, v/v), followed by chloroform, acetone, methanol, acetic acid and water (50:20:10:10:4, v/v) for the second direction. The separation of NBD-labeled lipids was obtained by one-dimensional thin layer chromatography using chloroform, ethanol, trimethylamine and water (30:35:35:7, v/v). NBD-labeled lipids were quantified on a ChemiDoc XR+ system with Image Lab software and Dylight 488 channel filter for Blue Epi illumination (Bio-Rad, Hercules, CA). Cellular lipids were visualized by Primuline staining (0.05% w/v in acetone:water, 8:2, v/v) using 365 nm UV light and quantitated by phosphate determination (Rouser et al., 1970). Phospholipids were identified by comparison with commercial phospholipid standards (Avanti Polar Lipids).

### Data analysis

Unless otherwise noted, all data are presented as mean±s.d. of at least three independent experiments (i.e. using three different cell preparations). For NBD-labeled lipid uptake experiments, data were fitted to a single-exponential curve with Microsoft Excel and the SOLVER add-in (Microsoft Corp., Redmond, WA) using the equation y=*A*+*B*(1−e^−*Ct*^), where *t* is the time after NBD-labeled lipid addition, *A* is the amount of non-extractable NBD-labeled lipid at *t*=0, *B* is the amount of non-extractable NBD-labeled lipid at steady state and *C* is the rate coefficient. The initial influx rate was derived from the product *B*×*C* and used consistently for presenting and comparing results. For multiple datasets, we used ANOVA and Tukey's honestly significant difference (HSD) tests to determine the variance in the experimental results obtained. Significance was accepted at *P*<0.05. Error bars are shown as mean±s.d.

## Supplementary Material

10.1242/joces.258649_sup1 Supplementary information
