# Supplementary material for: CDC50A is required for aminophospholipid transport and cell fusion in mouse C2C12 myoblasts
Source: J Cell Sci. 2021 Oct 19;135(5):jcs258649. doi: 10.1242/jcs.258649 (PMC10405909; doi:10.1242/jcs.258649)
Supplement: Supplementary information [file joces-135-258649-s1.pdf]

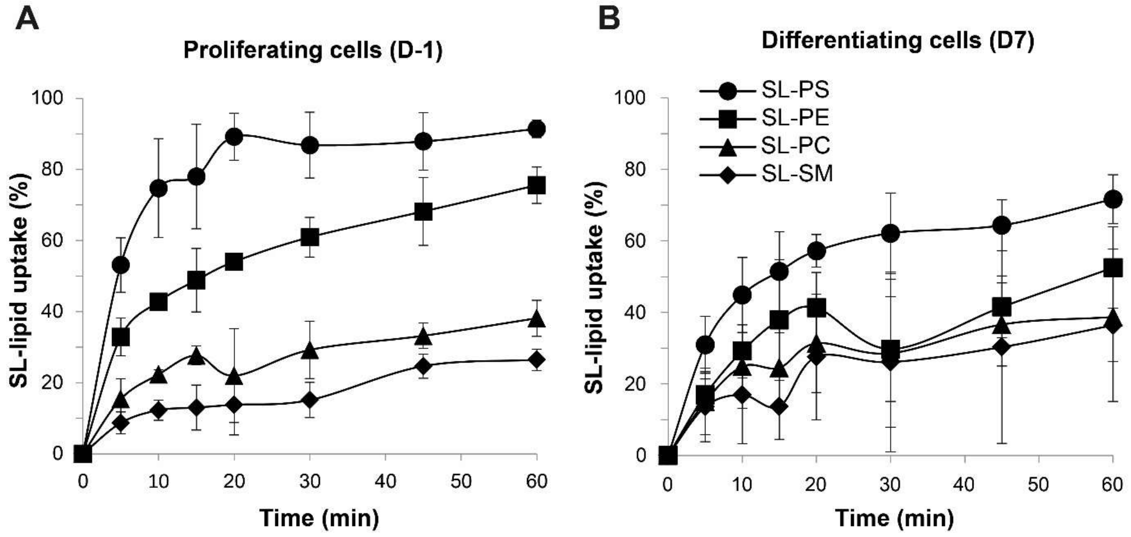


**Fig. S1. Internalization of spin-labeled lipids from the plasma membrane of proliferating and differentiating L6 rat skeletal muscle cells.** Internalization of the indicated spin-labeled (SL) lipids was analyzed during proliferation at D-1(A) and differentiation at day 7 (B) using back-exchange to albumin and expressed as the percentage of total label added at time zero. Data are mean ± S.D. from at least two (A) and three (B) independent experiments. PC, phosphatidylcholine; PE, phosphatidylethanolamine; PS, phosphatidylserine; SM, sphingomyelin.

Journal of Cell Science • Supplementary information

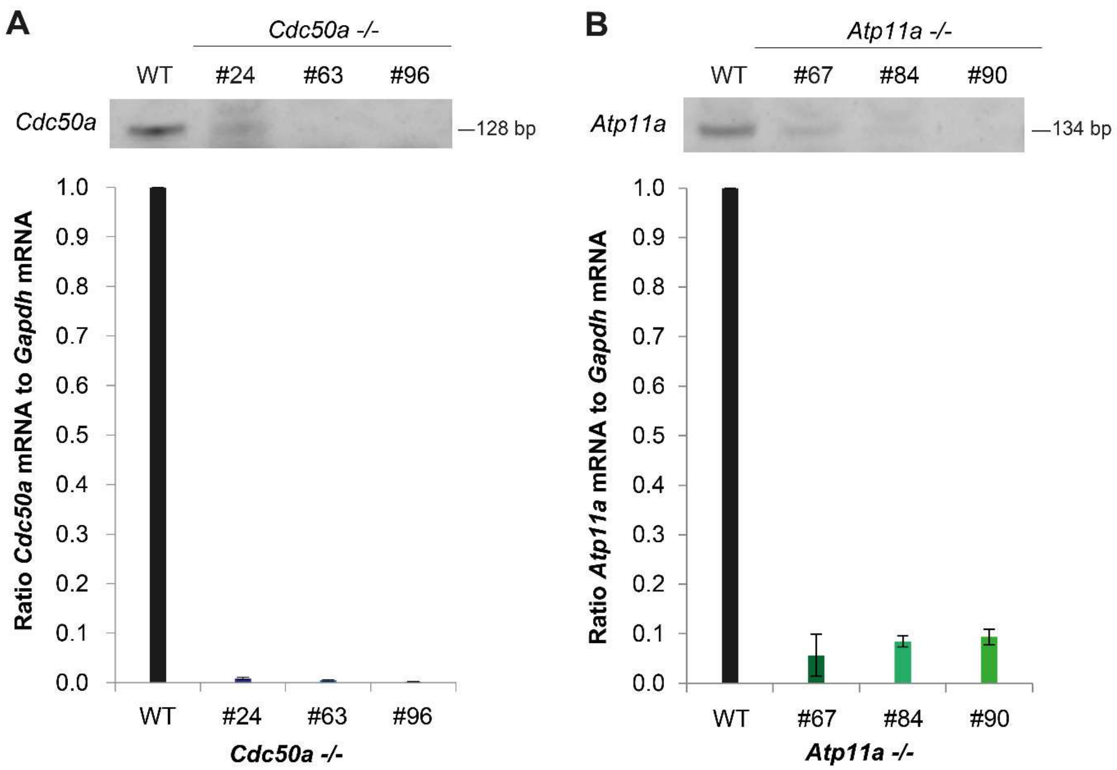


**Fig. S2. Validation of C2C12 knockout cell lines.** Analysis of the mRNA expression of *Cdc50a* and *Atp11a* transcripts in wild-type (WT) and clonal KO isolates by qRT-PCR. Ct values of genes of interest (GOI) were normalized to the housekeeping gene *Gapdh*. Top panels: agarose gel electrophoresis of the RT-PCR products. The experiment was carried out in duplicates and results shown are the mean ± S.D. of two independent experiments.

Journal of Cell Science • Supplementary information

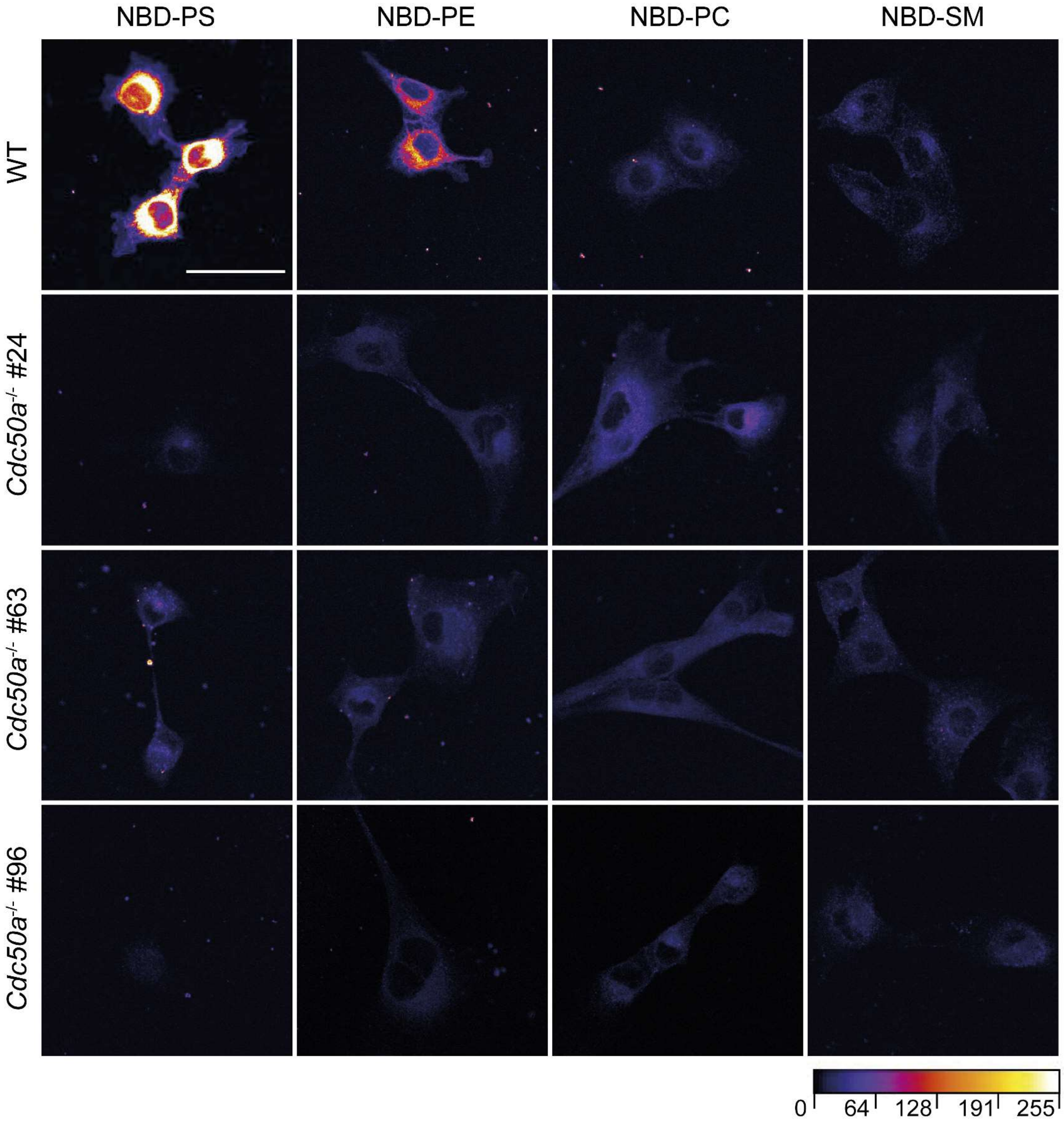


**Fig. S3. CDC50A-deficient C2C12 cells are impaired in aminophospholipid uptake.** Representative confocal images of proliferating wild-type (WT) and CDC50A-deficient cells (lines #24, #63 and #96) labeled with the indicated NBD-lipids for 60 min and subjected to back-exchange with albumin. Images were color-coded with the FIRE look-up-table to highlight intensity variations. In CDC50A-deficient cells, all NBD-lipids were hardly internalized and predominately detected in intracellular punctate structures. Scale bar, 50 µm. PC, phosphatidylcholine; PE, phosphatidylethanolamine; PS, phosphatidylserine; SM, sphingomyelin.

Journal of Cell Science • Supplementary information

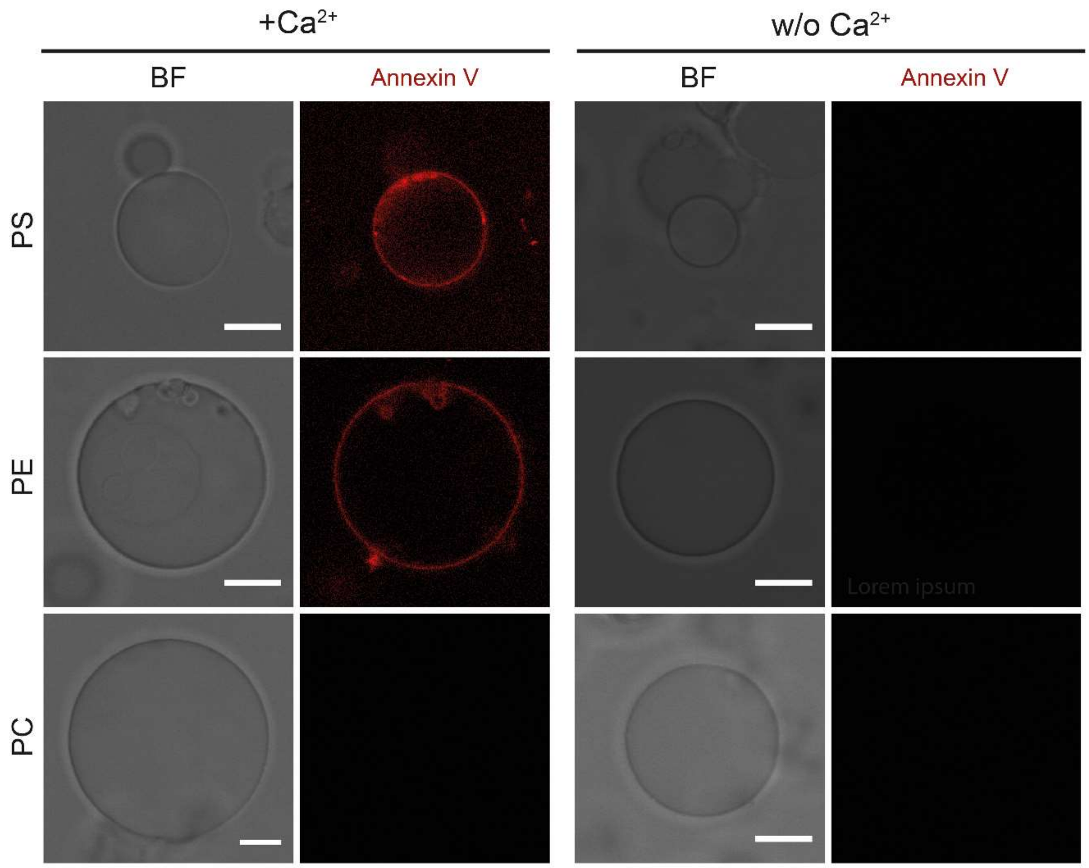


**Fig. S4. Annexin V-Alexa 568 binds PS and PE.** Giant unilamelar vesicles were prepared from different lipids and incubated with Annexin V-Alexa 568 (0.5 $\mu$L/mL) in the presence or absence of $Ca^{2+}$. Vesicles were analyzed by bright field (BF) and fluorescence microscopy. PC: PC only; PE: PC/PE, (9/1, mol/mol); PS: PC/PS, (9/1, mol/mol). Scale bar, 10 $\mu$m.

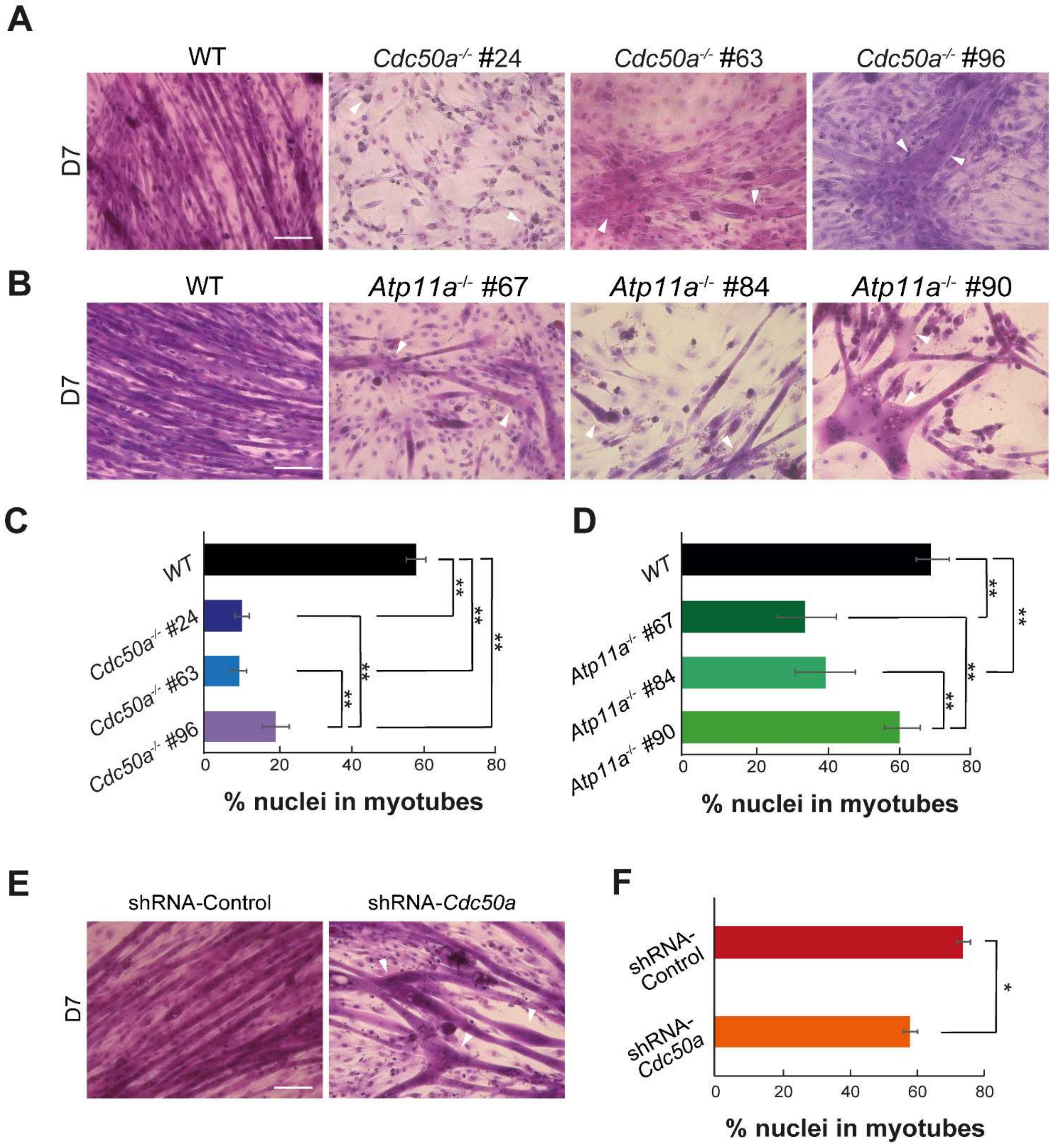


**Fig. S5. High passage C2C12 cells lacking CDC50A or ATP11A are affected in myotobe formation.** A, B, E) Representative microscopy images of wild-type (WT), three independently isolated CDC50A-deficient lines, three independently isolated ATP11A-deficient cell lines, and CDC50A-knockdown cells during differentiation at day 7 (D7). For knockdown, cells were stably transduced with scrambled shRNA control lentivirus or shRNA lentivirus targeted against CDC50A. Scale bars, 100 µm. Arrowheads indicate myotubes with abnormal morphology. C, D, F) Fusion index calculated at day 7 of differentiation. Data represents the mean ± S.D. of three experiments (ANOVA and TukeyHSD, **P<0.01).

Journal of Cell Science • Supplementary information

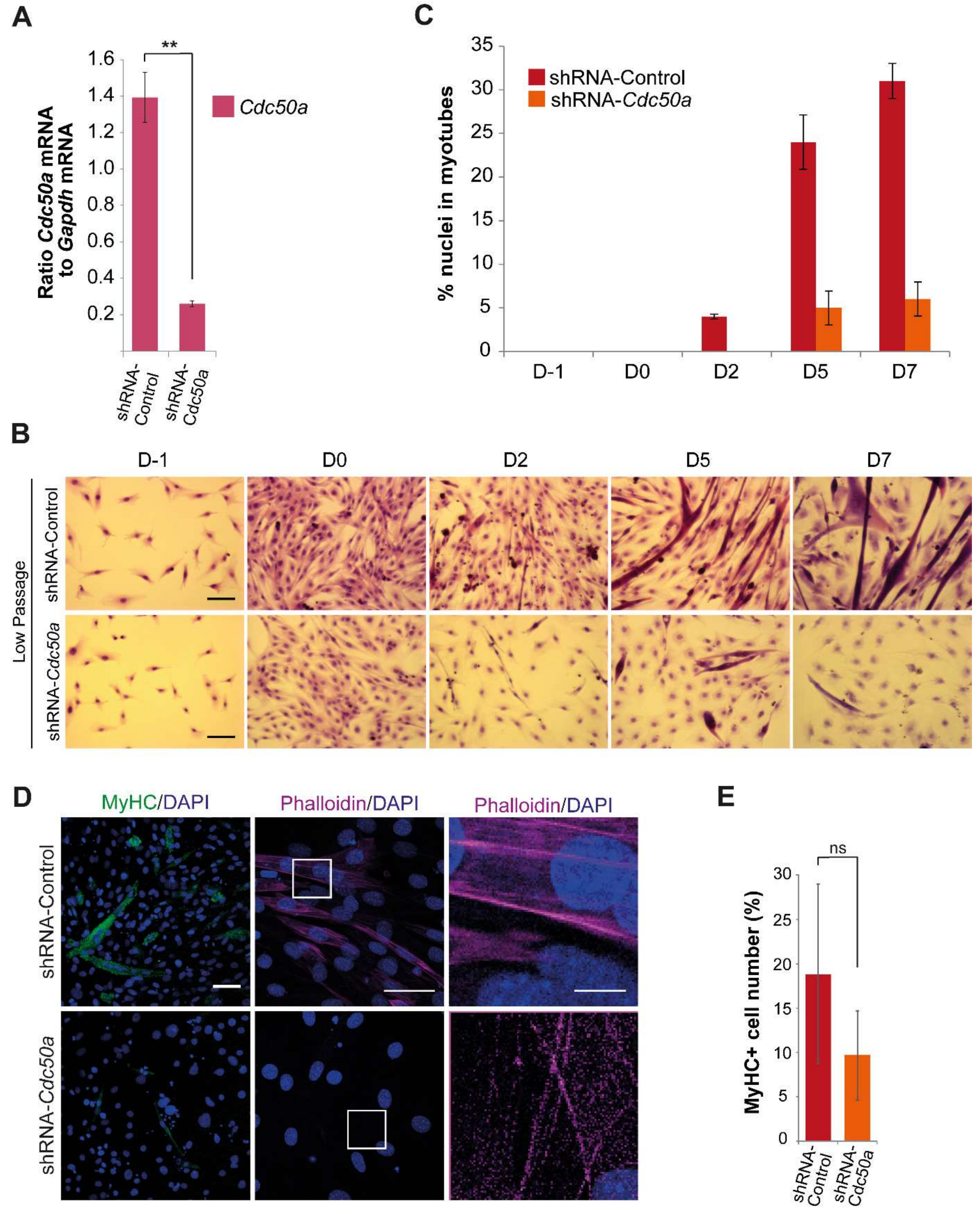


**Fig. S6. Cdc50a knockdown in C2C12 cells impairs myotube formation.** C2C12 cells were transduced with lentivirus carrying shRNA targeting CDC50A (shRNA-CDC50A) or the same amount of lentivirus carrying shRNA-Control. A) qRT-PCR was used to detect the efficiency of shRNA-*Cdc50a* mediated knockdown in proliferating C2C12 cells. The expression level of *Cdc50a* in the knockdown cells

Journal of Cell Science • Supplementary information

was reduced to ~20% of the level in shRNA-Control. Data are presented as mean ± S.D. of three experiments (ANOVA and TukeyHSD, **P < 0.01). B) Representative microscopy images of low passage control and CDC50A-knockdown cells during proliferation (D-1) and differentiation at days 0 (D0), 2 (D2), 5 (D5) and 7 (D7). Scale bar, 100 µm. C) Calculation of fusion indices showed that shRNA-Cdc50a cells were severely deficient in myotube formation. Data represents the mean ± S.D. of two distinct fields of one experiment. D) Confocal imaging of control and CDC50A-knockdown cells immunostained at day 2 of differentiation for MyHC (left panel, green) and F-actin (middle panel, phalloidin, red). Nuclei (DAPI) are in blue. Right panels show zoomed-in images of the white boxes, adjusted with different tonal values, brightness and contrast, in order to enhance the low signal intensities. Scale bars: 50 µm (left and middle panels), 10 µm (right panel). E) Percentage MyHC-positive cells at day 2 of differentiation. Bars indicate S.D. from the mean of two independent experiments (ANOVA and TukeyHSD; ns, not significant).

**Table S1.** Cell media and material

| Product | Company | Catalogue number |
|---|---|---|
| Dulbeccos's Modified Eagle's Medium - high glucose | Sigma-Aldrich | D6046-500ML |
| Dulbeccos's Modified Eagle's Medium - low glucose | Sigma-Aldrich | D6046-500ML |
| Fetal Bovine Serum | Capricorn Scientific | FBS-12A |
| Horse serum | Sigma-Aldrich | F9665-100ML |
| Penicilin/Streptomycin | Sigma-Aldrich | P0781-100ML |
| Trypsin | Sigma-Aldrich | T4549-100ML |
| Glutamin | Sigma-Aldrich | G7513-100ML |
| T-25 flasks | Sarstedt | 83.3910 |
| T-75 flasks | Sarstedt | 83.3911 |
| 35 mm Petri dishes | Ibidi | 81156 |
| Hanks' Balanced Salt Solution | Sigma-Aldrich | H6648-500ML |
| Duclbecco's Phosphate Buffered Saline | Sigma-Aldrich | D8537-500ML |
| Goat serum | SouthernBiotech | 0060-01 |
| DAPI | Sigma-Aldrich | D9524-5MG |
| Bovine Serum Albumin | Sigma-Aldrich | A7030-50G |
| Triton X-100 | Roth | 3051.3 |

Journal of Cell Science • Supplementary information

**Table S2.** Primers used for PCR

| Target | Type | Primer sequence | Amplicon size |
|---|---|---|---|
| *Cdc50a* WT.A | Fwd | GACCCTAGTGCTTTGCTTGTAAGT | 590 bp |
| | Rv | CACTATGGAAAGCTCAACAGTCAG | |
| *Cdc50a* HDR.A | Fwd | GTTGCTACTTAACCTCAGTAC | 463 bp |
| | Rv | CTACTGCACTTATATACGGTTC | |
| *Cdc50a* WT.C | Fwd | CCTATGCCTTCTCTGTCAGC | 306 bp |
| | Rv | CTTTTAGACAAGAAGGGATATTGATC | |
| *Cdc50a* HDR.C | Fwd | CTTCCATTTCTTGTGCTTAG | 387 bp |
| | Rv | GTGAAGGAGAGATGCGAG | |
| *Atp11a* WT.A | Fwd | GTCTCAACCTGACGGTGTTG | 504 bp |
| | Rv | GACTCCATCTCCACATCCTG | |
| *Atp11a* HDR.A | Fwd | GTCTCAACCTGACGGTGTTG | 387 bp |
| | Rv | GCACTTATATACGGTTCTCCC | |
| *Atp11a* WT.B | Fwd | GAAACATAAGGCAGCCTAAAC | 308 bp |
| | Rv | GGCAAACCTGCTTTGAAAAG | |
| *Atp11a* HDR.B | Fwd | GAAACATAAGGCAGCCTAAAC | 434 bp |
| | Rv | GCGAAAAAGAACGTTCACG | |
| *Atp11a* WT.C | Fwd | GTTGCTAGGTGTTTCCAGG | 408 bp |
| | Rv | GCCCTTAACTGTCAGACTACAC | |
| *Atp11a* HDR.C | Fwd | GTTGCTAGGTGTTTCCAGG | 416 bp |
| | Rv | GTGAAGGAGAGATGCGAGC | |

Journal of Cell Science • Supplementary information

**Table S3.** Primers used for RT-PCR

| Target | Type | Primer sequence | Amplicon size |
|---|---|---|---|
| *Atp8a1* | Fwd | TGAGAGATCGTGCAGAAAGGAG | 155 bp |
| | Rv | GGGCTTTCAGTGGGAACCAAAAC | |
| *Atp8a2* | Fwd | GCGTACTTCTGGTTGGGATTG | 166 bp |
| | Rv | CGTTACTGTCTCGAAGCATCG | |
| *Atp8b1* | Fwd | CAATTTTCGGCAGCATCGCACTG | 137 bp |
| | Rv | CAGCCAGATGTATGGCTGTCTC | |
| *Atp8b2* | Fwd | GACACAATCCTGCTAGACAGGC | 150 bp |
| | Rv | GGCCCACTCCTCATAGTACTCTTC | |
| *Atp8b3* | Fwd | ACTACGAGCCACTGGACATGC | 162 bp |
| | Rv | CGTCTGAATCATAGATGCAGCC | |
| *Atp8b4* | Fwd | GGCCACATCATTGGTCATTGTGG | 140 bp |
| | Rv | AGACACCATCACTGTGCATGGC | |
| *Atp8b5* | Fwd | CTAGTGCAGACGACTCTGATTGG | 166 bp |
| | Rv | AGATGCTGGGGTACCTGAGACAC | |
| *Atp9a* | Fwd | GGCCACTGTCCTACAAGACGTTC | 160 bp |
| | Rv | GCCACCATCAGTAGCTCAGTGAG | |
| *Atp9b* | Fwd | ACCCTGTTACGCCAGCATACC | 169 bp |
| | Rv | TGCCGATGTGTCTGAACTGTG | |
| *Atp10a* | Fwd | GTGGATGCTGCCTTTCAAAGCC | 138 bp |
| | Rv | AATACCCAGGTGCAGCAGGAAG | |
| *Atp10b* | Fwd | CAGTACCAATGTCTGTCCTTCCC | 146 bp |
| | Rv | CACCTCCCCACAGATCTTTGTCG | |
| *Atp10d* | Fwd | CCTCACCTTCTGGATCACCTTG | 161 bp |
| | Rv | CAATCACCAGGTGGAGGAGAATG | |

| | | | |
|---|---|---|---|
| *Atp11a* | Fwd | GCTGACCTTTGATTCTGTGCGG | 142 bp |
| | Rv | TCTAGACCGGACCTGGTCTAC | |
| *Atp11b* | Fwd | GGACCAGCTTATCTCTTGCACTCAG | 168 bp |
| | Rv | CATCACCAACAGCCAGTGTTATCGG | |
| *Atp11c* | Fwd | GAAACTGCTATTGGTTCATGGA | 143 bp |
| | Rv | CATAGAGTGGCTGTTGTGAG | |
| *Cdc50a* | Fwd | GCCAGTAAACTGGCATAAGGCAG | 159 bp |
| | Rv | GGGTGTAAATCATCTCTCCGCTC | |
| *Cdc50b* | Fwd | CTACCACGTCAAGTTCCGCAAC | 160 bp |
| | Rv | CATCCACACCACGAAGTCCTG | |
| *Cdc50c* | Fwd | CCTTCCGGAGAAAATGGAGGG | 173 bp |
| | Rv | TGATGGGAGTGTCATTGTGGG | |
| *Gapdh* | Fwd | CATCACTGCCACCCAGAAGACTG | 153 bp |
| | Rv | ATGCCAGTGAGCTTCCCGTTCAG | |

| | | | |
|---|---|---|---|
| Knockout analysis by RT-PCR | | | |
| *Cdc50a* mRNA | Fwd | GGCAGTATATGAGCTAGACCCTGA | 128 bp |
| | Rv | CTCTCCGCTCTATGAGACGATAC | |
| *Atp11a* mRNA | Fwd | CATATCCATGCTGTCCAGTGGG | 134 bp |
| | Rv | CATCGGCCAATGCTTTGAGTC | |
| Housekeeping gene | | | |
| *Gapdh* | Fwd | CATCACTGCCACCCAGAAGACTG | 153 bp |
| | Rv | ATGCCAGTGAGCTTCCCGTTCAG | |

Journal of Cell Science • Supplementary information
