# Supplementary material for: CDC50A is required for aminophospholipid transport and cell fusion in mouse C2C12 myoblasts
Source: J Cell Sci. 2021 Oct 19;135(5):jcs258649. doi: 10.1242/jcs.258649 (PMC10405909; doi:10.1242/jcs.258649)
Supplement: Supplementary file 1 [file joces-135-258649_review_history.pdf]

Journal of
Cell Science

CDC50A is required for aminophospholipid transport and cell fusion in mouse C2C12 myoblasts

Marta Grifell-Junyent, Julia F. Baum, Silja Väelimets, Andreas Herrmann, Coen C. Paulusma, Rosa L. López-Marqués and Thomas Güenther Pomorski
DOI: 10.1242/jcs.258649

Editor: James Olzmann

Review timeline
Original submission: 17 March 2021
Editorial decision: 26 April 2021
First revision received: 2 September 2021
Accepted: 2 September 2021

Original submission

First decision letter

MS ID#: JOCES/2021/258649

MS TITLE: CDC50A is required for aminophospholipid transport and cell fusion in mouse C2C12 myoblasts

AUTHORS: Marta Grifell Junyent, Julia Baum, Silja Vaelimets, Andreas Herrmann, Coen C. Paulusma, Rosa Laura Lopez Marques, and Thomas Guenther Pomorski
ARTICLE TYPE: Research Article

We have now reached a decision on the above manuscript.

To see the reviewers' reports and a copy of this decision letter, please go to: https://submit-jcs.biologists.org and click on the 'Manuscripts with Decisions' queue in the Author Area. (Corresponding author only has access to reviews.)

As you will see, the reviewers raise a number of comments that prevent me from accepting the paper at this stage. They suggest, however, that a revised version might prove acceptable, if you can address their concerns. In particularly, Reviewer 1's comments regarding why your results contrast with those from Tsuchiya et al (Nat Comm 2018) seem to be warranted and should be addressed, preferably with the proposed experiments comparing cell lines and conditions employed in the two studies. Reviewer 2 raises some concerns regarding interpretations and conclusions and the need to add statistical analyses to specific experiments. If you think that you can deal satisfactorily with the criticisms on revision, I would be pleased to see a revised manuscript. We would then return it to the reviewers.

We are aware that you may be experiencing disruption to the normal running of your lab that makes experimental revisions challenging. If it would be helpful, we encourage you to contact us to discuss your revision in greater detail. Please send us a point-by-point response indicating where you are able to address concerns raised (either experimentally or by changes to the text) and where you will not be able to do so within the normal timeframe of a revision. We will then provide further guidance. Please also note that we are happy to extend revision timeframes as necessary.

© 2021. Published by The Company of Biologists under the terms of the Creative Commons Attribution License (https://creativecommons.org/licenses/by/4.0/).

Please ensure that you clearly highlight all changes made in the revised manuscript. Please avoid using 'Tracked changes' in Word files as these are lost in PDF conversion.

I should be grateful if you would also provide a point-by-point response detailing how you have dealt with the points raised by the reviewers in the 'Response to Reviewers' box. Please attend to all of the reviewers' comments. If you do not agree with any of their criticisms or suggestions please explain clearly why this is so.

Reviewer 1

Advance summary and potential significance to field

Membrane remodeling appears to play a crucial role in the fusion of myoblasts to form multinucleated myofibrils. A previous study presented evidence that disruption of CDC50A and ATP11A, two subunits of a P4-ATPase that establishes PS asymmetry of the membrane, caused promiscuous fusion of myoblasts and syncytium formation rather than well-organized myofibrils. The current manuscript draws the opposite conclusion for CDC50A KO myoblasts (defect in fusion) and suggests a minimal role for ATP11A. If true, this would be a significant advance for the field.

Comments for the author

The manuscript by Junyent and colleagues analyzes the role of P4-ATPase mediated control of membrane organization on the fusion of C2C12 myoblasts. The approach is basically a repeat of the Tsuchiya et al (Nat Comm, 2018) paper but surprisingly, very different results were obtained. Whereas Tsuchiya and colleagues found that knocking out CDC50A or ATP11A caused excessive and unregulated cell fusion to form abnormally large syncytia, Junyent and colleagues find that knocking out CDC50A causes a loss of membrane fusion and the ATP11A KO does not appear to significantly influence cell fusion. I think it is essential that the authors do more to determine why such different results were obtained relative to a published study. The authors only say that the reason for the discrepancy is unclear, but it is not clear if any effort has been made to determine why the different results were obtained.

1. The authors should request the ATP11A and CDC50A deficient C2C12 myoblasts from the Umeda lab to examine side-by-side with the ATP11A/CDC50A cell lines they have created in the cell fusion assays. They should also ascertain if the media and differentiation conditions differ between the two studies and attempt to repeat the Tsuchiya experiments as they performed them.

2. Figure 3 shows very different P4-ATPase expression profiles for P4-ATPases relative to what is shown in the supplemental figure 1c of the Tsuchiya paper.
The basis for these differences could also be examined side-by-side with C2C12 cells from the Umeda lab to determine why Atp11c appears to be weakly expressed and Atp8b2 highly expressed in the authors' cells.

3. Figures 5 and 6 and their legends are scrambled in the submitted manuscript.
The in text call outs for these figures are also inaccurate.

4. The Phalloidin staining is not apparent at all in the Figure 6 (intended fig 5) images of Cdc50a cells. The text indicates a reduction in intensity and loss from the cortex. Perhaps an enlarged image taken at longer exposure could be included in the supplement to support the claim that the cortex/cytoplasm ration is perturbed.

Minor issues
5. For accuracy and consistency, the figure 1C Y-axis should read % of total phospholipid.

6. How were the transport assays in Figure 2A normalized? (% of maximum uptake for NBD-PS? If so, why does the NBD-PS plateau at 90% and not 100%?)

© 2021. Published by The Company of Biologists under the terms of the Creative Commons Attribution License (https://creativecommons.org/licenses/by/4.0/).

Reviewer 2

Advance summary and potential significance to field

This study explores the mechanisms that underlie a transient loss of lipid asymmetry of the plasma membranes of differentiating myoblasts fusing to form multinucleated myotubes. The work reports that lipid asymmetry in myoblasts is maintained by the activity of CDC50A-dependent P4-ATPases. While CDC50A is required for myotube formation, this CDC50A-dependent flippase activity is downregulated during myogenic differentiation. These findings are novel and important for the field and the main conclusion of the paper ("CDC50A is required for aminophospholipid transport and cell fusion in mouse C2C12 myoblasts") is well substantiated. However, several conclusions that the authors made seem to be premature or unfounded with results presented in the manuscript.

Comments for the author

1) The authors consider their finding that "C2C12 myoblasts hardly take up NBD-PC and NBD-SM but efficiently internalize NBD-PS and NBD-PE", as evidence against "the presence of an active scramblase in the plasma membrane of C2C12 cells during the proliferation and differentiation phases." However, in my opinion, the evidence for the conclusion that PS appearance at the surface of differentiating myoblasts can be fully explained by a lowered flippase activity and does not involve any scramblase activity is weak. To start with, there has been an earlier report that documents the importance of a scramblase (ANO5) in myoblast fusion (Whitlock et al., J Gen Physiol , 2018, 150, 1498) and must be cited/considered in this discussion. Furthermore, it appears that the authors expect the presence of an active scramblase to necessarily lead to a complete loss of lipid asymmetry. However, it seems to be a question of relative efficiencies of lipid transfer by scramblase and flippase: non-specific scrambling at the background of a suppressed but still working specific flippase can result in different rates of internalization for different lipids. Moreover as described in the Introduction, earlier studies reported "a transient exposure of PS in the outer leaflet of the plasma membrane of fusion-committed myoblasts at cell–cell contact sites". I am not sure the experimental approach used here
(flow cytometry analysis of lifted cells) would pick up a transient and localized scramblase-mediated disruption of lipid asymmetry.

2) The work reports downregulation of the flippase activity during differentiation. Is this downregulation required for fusion? This question can be addressed by testing whether overexpressing CDC50A will influence (inhibit?) fusion?

3) The conclusion "that C2C12 myoblasts are dependent on CDC50A for cell fusion" rather than for pre-fusion stages of myogenic differentiation is based on finding that some "CDC50A-deficient cells were still capable of expressing the late-stage differentiation marker myosin heavy chain (MyHC)". However, the fluorescence microscopy images in Figures 5 and S5 seem to show that only a very small fraction of CDC50A-deficient cells expresses MyHC. Furthermore, myoblast fusion requires both of two fusing myoblasts to be differentiated (express myomaker) (Millay et al., Nature, 2013, 499, 301-305). Taking this into account it is unclear why the authors conclude that fusion defect for CDC50A-deficient cells cannot be explained by the CDC50A-dependence of the prefusion stages.

4) The legend for the Fig 5 describes Fig 6 and the legend for the Fig 6 describes Fig. 5.

5) Some figures do not show relevant statistical analysis and/or the statistics is unclear. For instance, are any differences in Fig. 5C and Fig S5 statistically significant? Also, the legend states that each value "represents mean ± S.D. of two distinct fields of one experiment". Does it mean that this experiment was done only once? Moreover, data points in myoblast fusion studies are conventionally based on analysis of at least 5 random fields and/or many more than "A minimum of 100 nuclei " (see, for instance, Millay et al., Nature 2013, 499, 301-305).

6) I have found no explanation for labels "a" and "b" above bars in Figure 5C D2?

© 2021. Published by The Company of Biologists under the terms of the Creative Commons Attribution License (https://creativecommons.org/licenses/by/4.0/).

7) The statement "…large, morphologically abnormal multinucleated syncytia for ATP11A-deficient cells were rarely observed (< 22 ± 13% of the total myotubes)."

What clone was used in these experiments? What was the total number of myotubes in the analysis?
8) I have not found some of the references mentioned in the text (for instance Iri et al., 2017) in the list of references.

First revision

Author response to reviewers' comments

Dear Dr. Olzmann,

We would like to thank you and the reviewers for constructive comments and suggestions. We are submitting the revised version of the manuscript including Main Text and Supporting Information. In the revised version, we have modified the content according to the suggestions of the reviewers. Specifically, we have included additional experiments to evaluate potential reasons for the different results obtained in our study and that of Tsuchiya et al (Nat Comm, 2018), improved the statistical analysis of our data, and added a detailed source list of all culture material used in the Supporting Information. In addition, we included numerous text changes to clarify our points and to improve the Discussion. Please find below a detailed response to the reviewers' comments, as well as a revised version of our manuscript with all changes highlighted in blue font. With these changes, we hope that the manuscript can now be accepted for publications in the Journal of Cell Science.

Sincerely yours,

Thomas Günther-Pomorski

Point-by-point response to the referees' comments
We thank both reviewers for their helpful comments to our original manuscript.

Reviewer #1
Advance Summary and Potential Significance to Field
Membrane remodeling appears to play a crucial role in the fusion of myoblasts to form multinucleated myofibrils. A previous study presented evidence that disruption of CDC50A and ATP11A, two subunits of a P4-ATPase that establishes PS asymmetry of the membrane, caused promiscuous fusion of myoblasts and syncytium formation rather than well-organized myofibrils. The current manuscript draws the opposite conclusion for CDC50A KO myoblasts (defect in fusion) and suggests a minimal role for ATP11A. If true, this would be a significant advance for the field.

Comments for the Author
The manuscript by Junyent and colleagues analyzes the role of P4-ATPase mediated control of membrane organization on the fusion of C2C12 myoblasts. The approach is basically a repeat of the Tsuchiya et al (Nat Comm, 2018) paper but surprisingly, very different results were obtained.
Whereas Tsuchiya and colleagues found that knocking out CDC50A or ATP11A caused excessive and unregulated cell fusion to form abnormally large syncytia, Junyent and colleagues find that knocking out CDC50A causes a loss of membrane fusion and the ATP11A KO does not appear to significantly influence cell fusion. I think it is essential that the authors do more to determine why such different results were obtained relative to a published study. The authors only say that the reason for the discrepancy is unclear, but it is not clear if any effort has been made to determine why the different results were obtained.
Response: We essentially followed the experimental procedure based on the short description provided by Tsuchiya et al. (2018). As described by Tsuchiya et al. (2018), we maintained the

© 2021. Published by The Company of Biologists under the terms of the Creative Commons Attribution License (https://creativecommons.org/licenses/by/4.0/).

cells in growth medium consisting of Dulbecco's Modified Eagle's Medium (DMEM) supplemented with 20% of fetal calf serum. Myotube formation was induced by medium change to differentiation medium consisting of DMEM with 2% of horse serum. We have now added a detailed source list of all culture material used in our work in Suppl. Table S1. The C2C12 cell line we used was obtained from the same supplier (ATCC), however, we cannot exclude that the individual cell batches might differ from each other in many physical and biological aspects including passage history, medium and serum batch used for culture.
However, following the Reviewer's suggestion, we further tested different culture conditions, including the effects of temperature, plastic surface, cell density and the passage number of the cells. These additional experiments identified the passage number as one critical factor. With higher passages, the number of abnormal multinucleated syncytia increased for both CDC50A- and ATP11A- deficient cell clones. These new data are presented in Suppl. Figure S5, and we stress now the importance of cell passage number on cell fusion in the text.

1. The authors should request the ATP11A and CDC50A deficient C2C12 myoblasts from the Umeda lab to examine side-by-side with the ATP11A/CDC50A cell lines they have created in the cell fusion assays. They should also ascertain if the media and differentiation conditions differ between the two studies and attempt to repeat the Tsuchiya experiments as they performed them.
Response: While we appreciate the reviewer's comment, we would like to point out that we generated three independent CDC50A ko lines that all display similar phenotypes: a loss of aminophospholipid transport at the plasma membrane and a defect in cell fusion. In addition, we prepared CDC50A knockdown cells, which again showed a defect in cell fusion. In additional experiments we now have identified the passage number as a critical factor in cell fusion and as a possible explanation for the discrepancy with the Tsuchiya data. These new data are presented in Suppl. Figure S5.

2. Figure 3 shows very different P4-ATPase expression profiles for P4-ATPases relative to what is shown in the supplemental figure 1c of the Tsuchiya paper. The basis for these differences could also be examined side-by-side with C2C12 cells from the Umeda lab to determine why Atp11c appears to be weakly expressed and Atp8b2 highly expressed in the authors' cells.
Response: The differences observed in gene expression can be caused by a number of factors, including passage history, medium and serum batch used for culture. Based on our new results, cell batch and passage history are likely responsible for any differences, as reported for many other cell types.

3. Figures 5 and 6 and their legends are scrambled in the submitted manuscript. The in text call outs for these figures are also inaccurate.
Response: We apologize for this mistake, which is now corrected in the revised version.

4. The Phalloidin staining is not apparent at all in the Figure 6 (intended fig 5) images of Cdc50a cells. The text indicates a reduction in intensity and loss from the cortex. Perhaps an enlarged image taken at longer exposure could be included in the supplement to support the claim that the cortex/cytoplasm ration is perturbed.
Response: We thank the reviewer for this suggestion. Following the Reviewer's suggestion, ZOOM-In images adjusted with different brightness and contrast are now provided in Figure 6A to illustrate the changes in cortex labeling.

<u>Minor issues</u>

5. For accuracy and consistency, the figure 1C Y-axis should read % of total phospholipid.
Response: Thank you for this comment. Following the Reviewer's suggestion, the figure has been edited accordingly.

6. How were the transport assays in Figure 2A normalized? (% of maximum uptake for NBD-PS? If so, why does the NBD-PS plateau at 90% and not 100%?).
Response: Regarding Point 1, we apologize for not having explained the data normalization clearly enough. For each NBD lipid, the percentage of uptake was calculated by comparing the cell- associated fluorescence intensity before and after BSA treatment. BSA extraction results in

© 2021. Published by The Company of Biologists under the terms of the Creative Commons Attribution License (https://creativecommons.org/licenses/by/4.0/).

the removal of NBD-lipids from the outer leaflet of the cell plasma membrane. Thus, by comparing the fluorescence intensity before and after BSA treatment, it is possible to calculate the percentage of internalized NBD lipid that is not accessible to BSA. The percentage of uptake (U) for each fluorescent lipid is calculated as $U = (F_{BSA}/F_{buffer}) \times 100$, where F_{BSA} is the geometric mean fluorescence of the BSA-treated cells and F_{buffer} is the geometric mean fluorescence of the control cells not treated with BSA. This information has now been included in the Methods section.
Regarding Point 2, the lipid distribution across the plasma membranes results from a continuous inward and outward movement of lipids between the two monolayers by flippases and floppases, respectively. At steady-state, the NBD-PS plateau reached at 90% and not 100%. This is in line with previous reports for erythrocytes (Morrot et al., 1989, Biochemistry 28, 3456-3462; Colleau et al., 1991, Chem Phys Lipids 57, 29-37) and fibroblasts (Pomorski et al., 1996, J. Cell Sci. 109, 687-698).

Reviewer #2
Advance Summary and Potential Significance to Field
This study explores the mechanisms that underlie a transient loss of lipid asymmetry of the plasma membranes of differentiating myoblasts fusing to form multinucleated myotubes. The work reports that lipid asymmetry in myoblasts is maintained by the activity of CDC50A-dependent P4-ATPases. While CDC50A is required for myotube formation, this CDC50A-dependent flippase activity is downregulated during myogenic differentiation. These findings are novel and important for the field and the main conclusion of the paper (“CDC50A is required for aminophospholipid transport and cell fusion in mouse C2C12 myoblasts”) is well substantiated. However, several conclusions that the authors made seem to be premature or unfounded with results presented in the manuscript.

Comments for the Author
1) The authors consider their finding that “C2C12 myoblasts hardly take up NBD-PC and NBD-SM but efficiently internalize NBD-PS and NBD-PE“, as evidence against “the presence of an active scramblase in the plasma membrane of C2C12 cells during the proliferation and differentiation phases.” However, in my opinion, the evidence for the conclusion that PS appearance at the surface of differentiating myoblasts can be fully explained by a lowered flippase activity and does not involve any scramblase activity is weak. To start with, there has been an earlier report that documents the importance of a scramblase (ANO5) in myoblast fusion (Whitlock et al., J Gen Physiol , 2018, 150, 1498) and must be cited/considered in this discussion. Furthermore, it appears that the authors expect the presence of an active scramblase to necessarily lead to a complete loss of lipid asymmetry. However, it seems to be a question of relative efficiencies of lipid transfer by scramblase and flippase: non-specific scrambling at the background of a suppressed but still working specific flippase can result in different rates of internalization for different lipids. Moreover, as described in the Introduction, earlier studies reported “a transient exposure of PS in the outer leaflet of the plasma membrane of fusion-committed myoblasts at cell-cell contact sites”. I am not sure the experimental approach used here (flow cytometry analysis of lifted cells) would pick up a transient and localized scramblase-mediated disruption of lipid asymmetry.
Response: The reviewer is correct that our assay cannot pick up a transient and localized scramblase-mediated disruption of lipid asymmetry. Thus, we cannot exclude the presence of a scramblase activity based on our current data. We have revised the paragraph accordingly: “These data are consistent with the presence of an active aminophospholipid flippase activity at the plasma membrane of proliferating C2C12 cell. During differentiation of myoblasts into myotubes, internalization of NBD-PS and NBD-PE (but not of NBD-PC and NBD-SM) dropped, indicative for a downregulation of the aminophospholipid flippase activity. Similar results were obtained for L6 rat skeletal muscle cells (Suppl. Fig. S1), suggesting these findings are species-independent. Recent studies have in addition indicated an important role of lipid scramblases in the regulation of membrane lipid organization during myoblast fusion (Kim et al, 2017; Whitlock et al., 2018). Thus, downregulation of the aminophospholipid flippase activity accompanied by a local increase in phospholipid scramblase activity can explain the previously reported transient exposure of PS in the outer leaflet of the plasma membrane of fusion-committed myoblasts at cell-cell contact sites during myoblast differentiation (van den Eijnde et al., 2001; Kaspar and Dvorák, 2008; Jeong and Conboy 2011).”

© 2021. Published by The Company of Biologists under the terms of the Creative Commons Attribution License (https://creativecommons.org/licenses/by/4.0/).

2) The work reports downregulation of the flippase activity during differentiation. Is this downregulation required for fusion? This question can be addressed by testing whether overexpressing CDC50A will influence (inhibit?) fusion?
Response: As suggested by the reviewer, we tested the effect of overexpressing CDC50A in wild-type cells using recombinant lentiviruses encoding hemagglutinin (HA)-tagged CDC50A under control of the PGK promoter. As shown below (Figure 1), wild-type cells expressing CDC50A (wt+) grew well under proliferating growth conditions. However, when cultured under conditions that promote differentiation, cells started detaching and exhibited severe defects in forming multinucleated myotubes. Thus, overexpression of CDC50A is toxic for C2C12 cells upon triggering differentiation.
These experiments have not been included in the manuscript (but can upon the editor's wish).

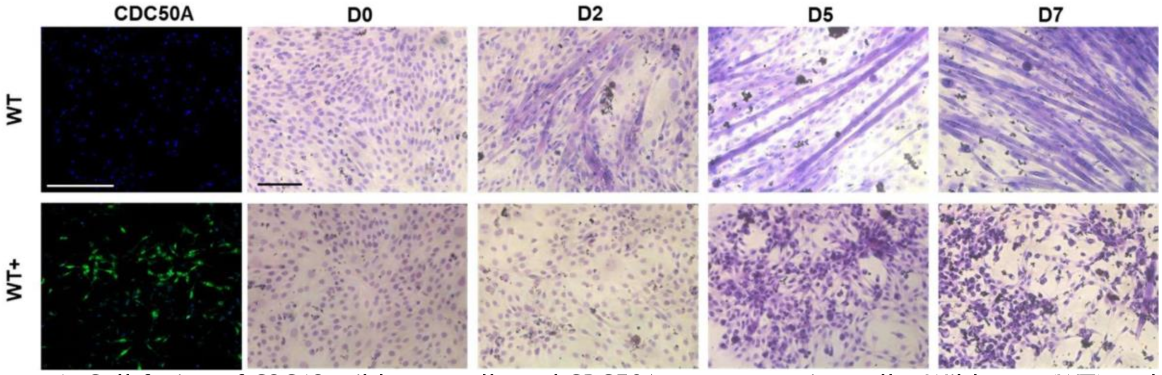

Figure 1: Cell fusion of C2C12 wild type cells and CDC50A overexpressing cells. Wild type (WT) and HA-tagged CDC50A overexpressing cells (WT+) were cultivated in growth medium and analysed by immunostaining (CDC50A) and multicolor staining (D0-7). At a confluence of 100% (day 0, D0), medium was changed to differentiation medium.

3) The conclusion "that C2C12 myoblasts are dependent on CDC50A for cell fusion" rather than for pre-fusion stages of myogenic differentiation is based on finding that some "CDC50A-deficient cells were still capable of expressing the late-stage differentiation marker myosin heavy chain (MyHC)". However, the fluorescence microscopy images in Figures 5 and S5 seem to show that only a very small fraction of CDC50A-deficient cells expresses MyHC. Furthermore, myoblast fusion requires both of two fusing myoblasts to be differentiated (express myomaker) (Millay et al., Nature, 2013, 499, 301-305). Taking this into account, it is unclear why the authors conclude that fusion defect for CDC50A-deficient cells cannot be explained by the CDC50A-dependence of the prefusion stages.
Response: The reviewer is correct. The MyHC expression is significantly reduced in CDC50A-deficient cells. We have revised the paragraph accordantly:
"The reduced ability of CDC50A-deficient cells to form multinucleated myotubes was paralleled by an interference with the biochemical differentiation, as revealed by examining the expression of the late-stage differentiation marker myosin heavy chain (MyHC). Analysis by immunofluorescence after 2 days in differentiation medium showed that CDC50A-deficient cells contained fewer MyHC positive cells than did equivalent control cultures (Fig. 5A, D and Suppl. Fig. S6D, E). Western blot analysis confirmed the lower expression of MyHC in CDC50A-deficient cells as compared to wild type (Fig.
5C). Thus, loss of CDC50A not only affects myoblasts in their competence to form multinucleated myotubes but also in their differentiation capacity."

4) The legend for the Fig 5 describes Fig 6 and the legend for the Fig 6 describes Fig. 5.
Response: We apologize for this mistake, which is now corrected in the revised version.

5) Some figures do not show relevant statistical analysis and/or the statistics is unclear. For instance, are any differences in Fig. 5C and Fig S5 statistically significant? Also, the legend states that each value "represents mean ± S.D. of two distinct fields of one experiment". Does it mean that this experiment was done only once? Moreover, data points in myoblast fusion studies are conventionally based on analysis of at least 5 random fields and/or many more than "A minimum of 100 nuclei " (see, for instance, Millay et al., Nature, 2013, 499, 301-305).

© 2021. Published by The Company of Biologists under the terms of the Creative Commons Attribution License (https://creativecommons.org/licenses/by/4.0/).

Response: We apologize that our presentation of statistical analysis in the first version of the manuscript was unclear. For each figure, we have now included a statistical analysis of the data along with the number of experiments performed. For the determination of the fusion index, the number of nuclei in each myotube (≥3 nuclei) and the total number of nuclei in cells were counted in three fields of at least two independent culture flasks for each experimental condition and time point. This analysis included >600 nuclei, except for day -1 (>100 nuclei) due to the low cell number at this culture stage. This information has been now included in the Methods section.

6) I have found no explanation for labels “a” and “b” above bars in Figure 5C D2?
Response: We thank the reviewer for this comment. In the revised version of the manuscript, we are now clearly indicating the statistical differences of the data.

7) The statement “…large, morphologically abnormal multinucleated syncytia for ATP11A-deficient cells were rarely observed (< 22 ± 13% of the total myotubes).” What clone was used in these experiments? What was the total number of myotubes in the analysis?
Response: As requested by the reviewer, this information is now provided. Morphologically abnormal multinucleated syncytia for low passage ATP11A-deficient cells were observed for less than half of the cells at day 7 (i.e. 58 out of 352 myotubes for clone 67; 56 out of 222 myotubes for clone 84; 115 out of 287 myotubes for clone 90).

8) I have not found some of the references mentioned in the text (for instance, Iri et al., 2017) in the list of references.
Response: This has been corrected in the revised version – thank you.

<u>Second decision letter</u>

MS ID#: JOCES/2021/258649

MS TITLE: CDC50A is required for aminophospholipid transport and cell fusion in mouse C2C12 myoblasts

AUTHORS: Marta Grifell Junyent, Julia Baum, Silja Vaelimets, Andreas Herrmann, Coen C. Paulusma, Rosa Laura Lopez Marques, and Thomas Guenther Pomorski
ARTICLE TYPE: Research Article

I have reviewed the changes you made in response to the reviewers' comments. I am happy to tell you that your manuscript has been accepted for publication in Journal of Cell Science, pending standard ethics checks. Thank you for submitting this interesting work to JCS!

© 2021. Published by The Company of Biologists under the terms of the Creative Commons Attribution License (https://creativecommons.org/licenses/by/4.0/).
